# Dietary and developmental shifts in butterfly-associated bacterial communities

**DOI:** 10.1098/rsos.171559

**Published:** 2018-05-30

**Authors:** Kruttika Phalnikar, Krushnamegh Kunte, Deepa Agashe

**Affiliations:** National Centre for Biological Sciences (NCBS), GKVK Campus, Bellary Road, Bangalore 560065 India

**Keywords:** microbiome, metamorphosis, insect, diet

## Abstract

Bacterial communities associated with insects can substantially influence host ecology, evolution and behaviour. Host diet is a key factor that shapes bacterial communities, but the impact of dietary transitions across insect development is poorly understood. We analysed bacterial communities of 12 butterfly species across different developmental stages, using amplicon sequencing of the 16S rRNA gene. Butterfly larvae typically consume leaves of a single host plant, whereas adults are more generalist nectar feeders. Thus, we expected bacterial communities to vary substantially across butterfly development. Surprisingly, only few species showed significant dietary and developmental transitions in bacterial communities, suggesting weak impacts of dietary transitions across butterfly development. On the other hand, bacterial communities were strongly influenced by butterfly species and family identity, potentially due to dietary and physiological variation across the host phylogeny. Larvae of most butterfly species largely mirrored bacterial community composition of their diets, suggesting passive acquisition rather than active selection. Overall, our results suggest that although butterflies harbour distinct microbiomes across taxonomic groups and dietary guilds, the dramatic dietary shifts that occur during development do not impose strong selection to maintain distinct bacterial communities across all butterfly hosts.

## 1. Introduction

All animals are associated with bacterial communities, and this association can significantly affect host biology [1–4]. In particular, insect models have been instrumental in understanding the mechanisms by which bacterial partners influence host physiology [5–7]. Bacteria can provide various benefits to insects, including efficient digestion, nutrient supplementation and detoxification; and can thus help insects to survive on suboptimal diets and occupy diverse dietary niches [7–13]. Through their impact on nutrient acquisition, bacteria can significantly alter traits such as host fecundity, survival and longevity [8,14–17]. In some insects, bacteria also influence other aspects of host physiology such as development, the immune system regulation, hormone signalling and resistance against infections [7,15,18–20]. Furthermore, bacteria can affect the behavioural ecology of insects by influencing mate choice and social aggregation via pheromone production [21,22]. Thus, to better understand insect ecology, evolution and behaviour, it is important to determine the factors that affect insect–bacterial associations.

Diet is one of the key factors that shape bacterial community structure across insect taxa [23–25]. For instance, gut microbiota of the omnivorous cockroach *Blattella germanica* is strongly affected by dietary protein content [26], and bacterial communities of *Drosophila melanogaster* vary with yeast, sugar and ethanol content in their diet [27]. A few studies also suggest that diet similarity is associated with convergent evolution of gut bacterial communities. For example, bacterial communities of detritivorous termites are more similar to those of detritivorous beetles and flies, rather than closely related wood-eating termites [23]. By contrast, in some cases diet does not significantly influence bacterial communities. For instance, communities of carnivorous and herbivorous larvae of different butterfly species are similar [28], as are communities associated with the adults and larvae of emerald ash borers that feed on foliage versus cambium [23]. Overall, diet has a variable but typically significant impact on bacterial communities of insects.

Dietary shifts occur not only across different insect taxa, but also within the lifetime of many insects including butterflies and moths (Lepidoptera), flies and mosquitoes (Diptera) and parasitoid wasps (Hymenopterans). In these orders, juvenile stages usually consume a distinct resource (typically solid) compared to the adult diet (typically liquid). These within-lifetime dietary switches are often associated with major developmental transformations. How do developmental transitions affect bacterial communities in these insects? Previous studies have found variable impacts of developmental stage on gut bacterial communities [29–35]. However, a comprehensive analysis incorporating multiple crucial factors in one study is still missing. These factors include multiple host developmental stages, individual variation between hosts, multiple host species to evaluate whether the results are generalizable, analysis of host diet to examine the route of bacterial acquisition, and analyses of wild-caught insects. Here, we attempt to fill these gaps using butterflies as a model.

Butterflies undergo complete metamorphosis involving four distinct developmental stages: egg, larva, pupa and adult. The dietary switch across stages is very stark in butterflies because larvae feed strictly on a solid diet, and adults only feed on liquids. The intermediate, non-feeding pupal stage is associated with massive tissue restructuring and physiological changes involving apoptosis and autophagy [36,37]. During this stage, the larval gut is degraded and the adult gut is formed anew [38,39]. Shortly after the adult butterfly ecloses from the pupa, it excretes metabolic waste, called meconium, generated in the pupal stage. Thus, within a butterfly's lifespan, both diet and physiology undergo a major transition. In the butterfly *Heliconius erato*, bacterial communities vary significantly across development, with only few bacterial phylotypes shared between larvae and adults [40]*.* However, it is unclear whether similar patterns occur across butterfly species, given the immense diversity in their diet, behaviour and life history. In particular, adult *Heliconius* butterflies feed on pollen, which is a unique dietary habit that is not observed in other butterflies [40]. Thus, the bacterial dynamics observed in this group may not reflect those of butterflies in general.

We used amplicon sequencing of the 16S rRNA gene to characterize the bacterial community structure of 12 butterfly species from five of the six described butterfly families: Nymphalidae, Lycaenidae, Pieridae, Papilionidae and Hesperiidae. Of these, we were able to analyse multiple individuals from different developmental stages of nine species (table 1). We also analysed bacterial communities associated with the larval diets of five of these species to test whether larval bacterial communities are largely diet-derived (table 1). We expected to find significant developmental changes in the bacterial community structure of each butterfly species, given the large differences in larval versus adult diet of each host (table 1). We also expected to find significant species-specific variation in bacterial communities associated with larvae, because each species has a distinct larval diet and caterpillars usually use a single host plant during development. On the other hand, we predicted that bacterial communities across adults would be more similar because they are known to be generalists—multiple butterfly species can feed on similar resources while foraging [41,42].

**Table 1.** Butterfly sampling design. The table shows the number of replicates analysed per developmental stage of each butterfly host species (E, egg; L, larva; P, pupa; A, adult). NNS-A, NNS-P and NNS-M correspond to Non-Nectar Sources (NNS) used by butterfly adults. NNS-M: adults obtain nutrients from mud fluids via mud puddling. In most cases, males tend to mud puddle and not females. Thus, mud puddling may not be a prominent dietary resource for most adults in these samples as most are females. NNS-P: adults feed on plant products such as plant sap or rotting plant tissues (e.g. flowers and fruits). NNS-A: adults feed on animal-derived resources, such as scat fluids, urine and sweat. All samples were collected from the same geographical location (NCBS campus and surrounding area, Bangalore) except samples of *P. brassicae* (Eaglenest Wildlife Sanctuary, Arunachal Pradesh).

	developmental stages					
butterfly species	E	L	P	A	larval diet	adult diet
family Lycaenidae						
*Spalgis epeus*		5	6	5	mealybugs—*Maconelicocus hirsutus* (Pseudococcidae)^a^	honeydew
*Pseudozizeeria maha*		—	—	3	—	nectar
*Jamides celeno*		—	—	3	—	nectar, NNS-A, NNS-M
*Leptotes plinius*		4	—	5	leaves/pods of *Plumbago zeylanica* (Plumbaginaceae)	nectar, NNS-A, NNS-M
family Nymphalidae						
*Ariadne merione*	—	5	5	5	leaves of *Ricinus communis* (Euphorbiaceae)^a^	nectar, NNS-P
*Danaus chrysippus*		2	3	3	leaves of *Calotropis gigantea* (Apocynaceae)^a^	nectar
*Elymnias caudata*		5	—	3	leaves of *Dypsis lutescens* (Arecaceae)^a^	NNS-A, NNS-P, NNS-M
family Pieridae						
*Pieris brassicae*		5	5	5	leaves of *Brassica* sp. (Brassicaceae)	nectar
*Eurema blanda*		4	7	5	leaves of *Albizia* sp. (Fabaceae)	nectar, NNS-A, NNS-M
family Hesperiidae						
*Erionota torus*		4	—	—	leaves of *Ensete superbum* (Musaceae)	nectar
*Gangara thyrsis*	1	5	8	2	leaves of *Dypsis lutescens* (Arecaceae)	nectar
family Papilionidae						
*Papilio polytes*	—	3	3	1	leaves of *Citrus* sp. (Rutaceae)^a^	nectar, NNS-A, NNS-M

^a^Cases where we also analysed bacterial communities associated with the larval diet.

Our work provides the first comprehensive analysis of bacterial communities across developmental stages of multiple wild-caught butterflies from different families and varied dietary habits. Unexpectedly, we find that within-species dietary shifts across larvae and adults have a weak impact on bacterial communities of butterflies. Similarly, the developmental transition during the pupal stage significantly affects bacterial community structure in some, but not all butterfly hosts. Thus, we find variable and generally weak impacts of butterfly development on bacterial communities. On the other hand, host taxonomy (species and family) significantly influences bacterial community structure, potentially owing to divergent dietary guilds or host physiology. Finally, in contrast to our expectation, we find that this impact of host taxonomy is stronger for butterfly adults compared to larvae.

## 2. Material and methods

### 2.1. Sample collection and sequencing

We collected butterfly samples and larval dietary resources in and around the campus of the National Centre for Biological Sciences, Bangalore (NCBS) (13.0714° N, 77.5802° E) from June 2014 to January 2016, and from the Eaglenest Wildlife Sanctuary, Arunachal Pradesh (27°06′0^″^ N, 92°24′0^″^ E) in June 2015. For collecting samples from Eaglenest Wildlife Sanctuary, we obtained collection permits from the state forest department of Arunachal Pradesh, India (permit no. CWL/G/13(95)/2011-12/Pt-III/2466-70). Information on butterfly identification, diets and larval host plants was taken from the Butterflies of India website (www.ifoundbutterflies.org/). We stored insects in 70% ethanol at −20°C until DNA extraction. We placed larvae and pupae in ethanol, and clipped off adult wings before storing the body. We surface-sterilized insect bodies by rinsing with fresh 70% ethanol and then 10% bleach for 30 s, each followed by three washes with sterile distilled water. We ground each sample in liquid nitrogen with single-use, sterilized pestles to homogenize the tissue. From the homogenized tissue, we extracted DNA using the Wizard^®^ Genomic DNA Purification Kit (Promega) according to the manufacturer's protocol. We dissolved the extracted DNA in sterile, DNAse/RNase free water and quantified DNA concentration using NanoDrop (NanoDrop 2000, Thermo Fisher Scientific Inc., Wilmington, USA). We outsourced library preparation and Illumina sequencing to Genotypic Technology Pvt Ltd, Bangalore, India. We sequenced the V3-V4 hypervariable region of the 16S rRNA gene using 300 or 250 bp paired-end sequencing on the Illumina MiSeq platform.

We followed the standard protocol for 16S rRNA gene sequencing (including 16S PCR primers, PCR conditions and library preparation specifications) by Illumina (https://support.illumina.com/documents/documentation/chemistry_documentation/16s/16s-metagenomic-library-prep-guide-15044223-b.pdf). Raw sequencing files (fastq) for all the samples are deposited in the European Nucleotide Archive (EMBL-EBI) database, under accession number PRJEB21255. We also tested for possible contamination from sources such as DNA extraction kits, as described in previous reports [43]. However, we did not find any evidence of contamination from our DNA extraction kits or library preparation kits (electronic supplementary material, methods).

### 2.2. Data processing and analysis

We analysed de-multiplexed MiSeq data using QIIME (version 1.9.1) [44]. We quality filtered reads with minimum quality score of q20. We removed chimaeric sequences using USEARCH (version 6.1) [45] and assembled filtered reads into Operational Taxonomic Units (OTUs) with 97% sequence similarity using UCLUST in QIIME. We picked OTUs with the ‘open reference OTU picking’ method in QIIME. After clustering reads into OTUs, one sequence from each OTU was used as a representative sequence. We mapped representative sequences to the Green Genes 16S ribosomal gene database (Greengenes Database Consortium, version gg_13_5) to assign taxonomy using default QIIME parameters. We removed OTUs categorized as mitochondria and chloroplast prior to analysis (electronic supplementary material, figure S1). After obtaining the number of reads for each OTU in each sample, we independently implemented five different cut-offs to remove rare bacterial OTUs from our analysis that may not contribute significantly to the bacterial community, or may have arisen due to PCR or sequencing errors (see the electronic supplementary material, methods for details). These cut-offs are as follows:

1. We selected only the five most abundant OTUs for each sample, henceforth ‘Top 5 OTUs cut-off’
2. We eliminated OTUs that had less than 20 reads from each sample, henceforth ‘20 read cut-off’
3. We eliminated OTUs that had less than 5% relative abundance in all the samples in our dataset, henceforth ‘5% abundance cut-off’
4. We eliminated OTUs that contributed less than 0.005% of the total reads across all samples, henceforth ‘0.005% abundance cut-off’
5. We extracted core OTUs that occur in at least 80% of all samples (OTU frequency based cut-off).

We considered all five rare-OTU filtering cut-offs independently for eliminating rare OTUs; however, we primarily focused on the two most conservative top 5 OTUs cut-off and 5% abundance cut-off for our main analysis and results. We selected these abundant bacterial OTUs in order to capture the dynamics of potentially impactful community members. Another reason to restrict our analysis to dominant bacterial OTUs (top 5 OTUs and 5% abundance cut-off) was the inconsistency in read depth observed across sequencing runs of Illumina. Out of 130 samples, 42 samples were sequenced in one run and 88 samples were sequenced in a second flow cell. We found substantial variation in read depths in samples that were processed in these two independent rounds of Illumina sequencing, with large differences in the total number of OTUs found in replicate samples of the same host species analysed in different sequencing runs (electronic supplementary material, figure S2). We suspect that the difference in OTU richness is due to differential sequencing depths and does not represent biologically relevant variation. To overcome this technical problem, we focused primarily on dominant bacterial OTUs. For each sample, the top 5 OTUs constituted a large proportion of the total bacterial community (average 87%, ranging from 66% to 99%; electronic supplementary material, figure S3). Similarly, with the 5% abundance cut-off we obtained comparable richness and composition of bacterial OTUs per sample across the two Illumina runs (electronic supplementary material, figure S2).

We characterized the bacterial communities associated with 120 butterfly samples from different families and varied dietary habits, and 10 samples of larval dietary resources (table 1). We collected multiple developmental stages for nine butterfly species and a single developmental stage for three species. All adult butterflies were females except for three adults of *S. epeus* for which the sex was unknown and one male adult of *E. caudata*. After quality filtering data in QIIME (described above), we obtained a total of 2.6 × 10^7^ reads and 70 348 OTUs across all 130 samples, with an average of 2 × 10^5^ reads and 1931 OTUs per sample. We define OTUs as clusters of reads that have at least 97% sequence identity, and discuss all our results in terms of OTUs rather than taxonomically identifiable units. After applying the five different rare-OTU filters (top 5 OTU cut-off, 5% abundance cut-off, 0.005% abundance cut-off, minimum 20 read cut-off and core OTUs), the total number of OTUs reduced to 5–13, 98, 964, 11 364 and 12 OTUs, respectively. In the analysis presented here, we focus on the two main cut-offs, 5% abundance cut-off and top 5 OTUs cut-off (mentioned in each section as applicable). However, the statistical analysis is presented for the OTU filtering cut-offs.

To identify the most abundant and frequent OTUs across butterfly host and diet samples, we used the OTU subset obtained by applying the 5% abundance cut-off. For this subset, we obtained an average of 1.3 × 10^5^ reads and 43 OTUs per butterfly sample (ranging from 14 to 67 OTUs; electronic supplementary material, figure S4). To characterize the most frequent bacterial OTUs across all butterfly samples, we calculated the proportion of samples that harboured each bacterial OTU, and categorized all OTUs that were present in more than or equal to 80% of the butterfly samples as ‘frequent OTUs’. Similarly, to determine the ‘most abundant OTUs’, we calculated the relative number of reads contributed by each OTU (i.e. relative abundance) in each sample, and then calculated the average relative abundance of a given OTU across all samples. In addition, we identified the five most abundant bacterial phyla, classes, orders and families by calculating the proportion of reads contributed by the respective taxon out of the total reads across all butterfly samples.

We used R for all statistical analyses [46]. We tested for variation in bacterial communities across (i) host development, (ii) host species, (iii) host families, and (iv) host diet. For comparing bacterial communities across these groups, we used permutational multivariate ANOVA (PERMANOVA) using the Adonis function in the R package Vegan [47]. To test the impact of developmental stage, host species and host family on bacterial community composition, we used canonical analysis of principal coordinates based on discriminant analysis (CAPdiscrim) and the function Ordiellipse in the R package BiodiversityR (electronic supplementary material, methods). CAPdiscrim performs constrained ordination based on Bray–Curtis distances. In addition, we performed an unconstrained analysis of principal coordinates (PCoA) using phylogenetic distance (weighted unifrac) to test whether bacterial OTUs present across butterfly species and families are phylogenetically distinct (electronic supplementary material, methods).

## 3. Results

### 3.1. Butterfly microbiomes are dominated by the genus *Wolbachia* and families Methylobacteriaceae and Enterobacteriaceae

**Figure 1.**
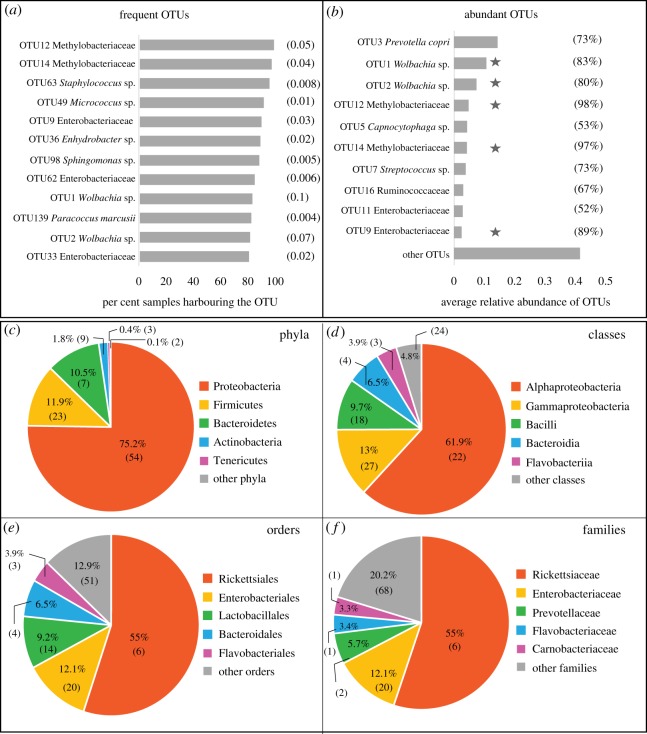
The most frequent and most abundant bacterial OTUs associated with butterflies. (*a*) The most frequent bacterial OTUs observed in more than 80% of butterfly samples. Numbers in parentheses represent the average relative abundance of each OTU. (*b*) The 10 most abundant bacterial OTUs (with the highest average relative abundance) across butterfly samples. Numbers in parentheses show the proportion of butterfly samples that harboured each abundant OTU. OTUs highlighted by a star are both abundant and frequent OTUs. (*c–f*) The five most abundant bacterial taxa across all butterfly samples. Each pie chart shows a different taxonomic rank, with each slice representing the percentage of total reads contributed by the taxon. Numbers in parentheses indicate the number of OTUs within each bacterial taxon.

The most frequent and abundant bacterial OTUs found across all butterfly samples belonged to the genus *Wolbachia,* family Enterobacteriaceae and family Methylobacteriaeceae (figure 1*a,b*). The most abundant bacterial phyla were Proteobacteria, Firmicutes, Bacteroidetes, Actinobacteria and Tenericutes (figure 1*c*). We observed that most of the bacterial classes, orders and families that dominated our focal butterfly species (figure 1*d*–*f*) were similar to those previously described for Lepidopterans [28,29,40,48,49] and other insects [23,24]. Next, we tested whether these patterns of bacterial occurrence and abundance showed significant developmental and host-associated variation.

### 3.2. Bacterial communities typically do not differ across butterfly development

**Figure 2.**
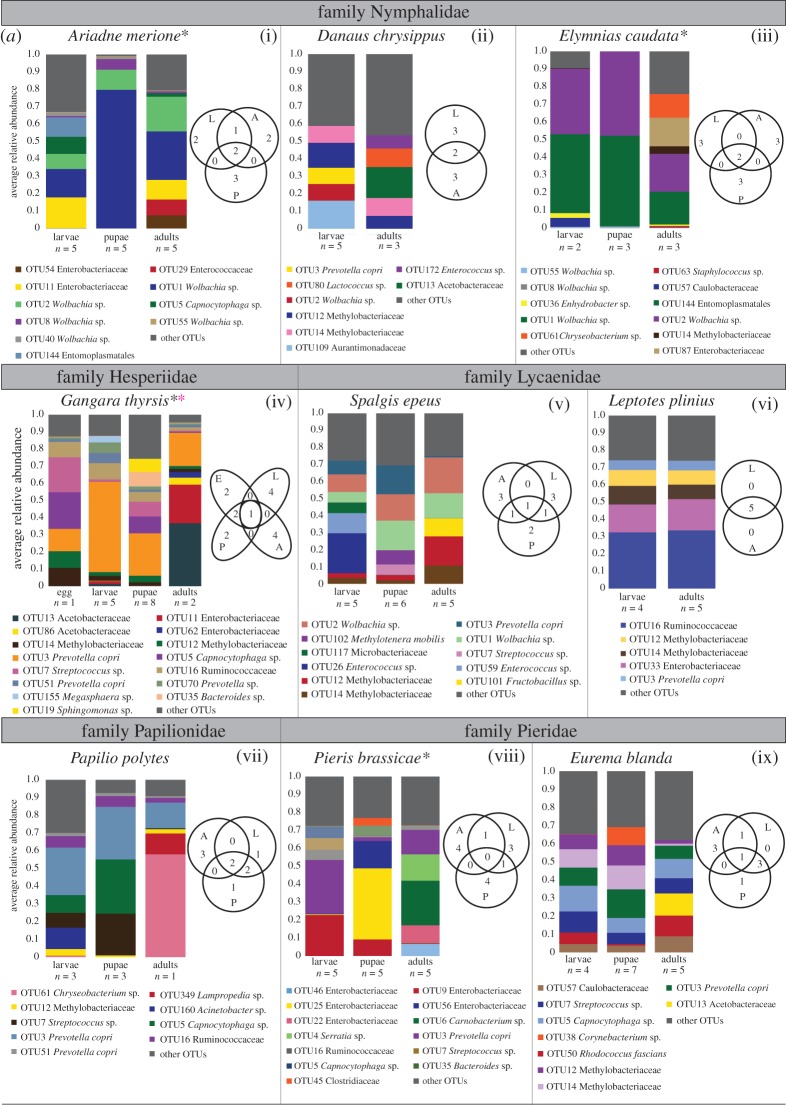

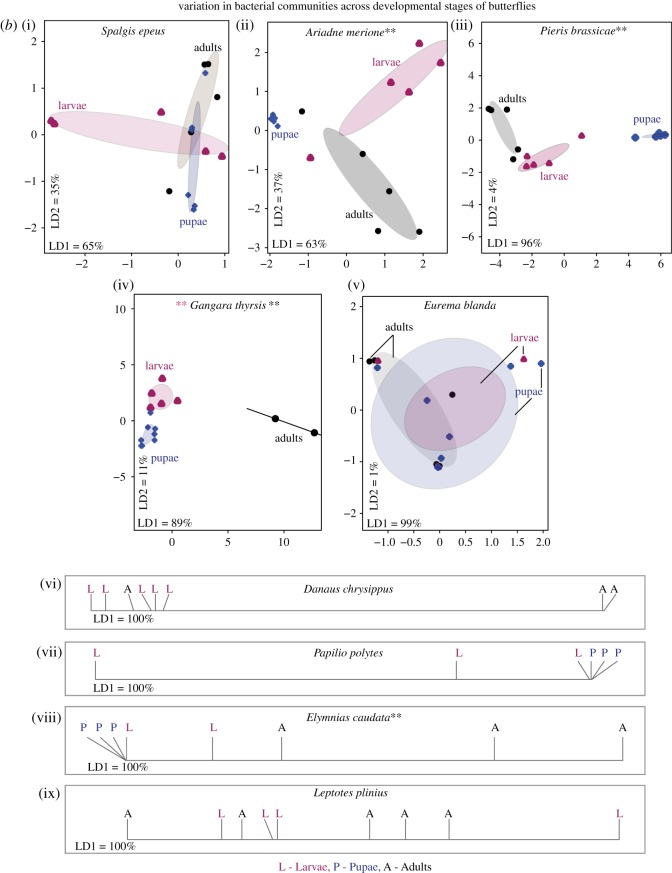
(*a*) Variation in dominant bacterial communities across butterfly life stages. Stacked bar plots show the average relative abundance of the top 5 dominant bacterial OTUs across all developmental stages of a host. Each panel shows data for a single butterfly species; panels are grouped by family. Venn diagrams in each panel show the number of dominant OTUs that are unique to each developmental stage. Black asterisks next to butterfly species names indicate significant variation in bacterial communities across all developmental stages (permutational multivariate ANOVA–PERMANOVA with 10 000 permutations; *p* < 0.05). Pink asterisks indicate significant variation in bacterial communities between larvae and adults. (*b*) Variation in bacterial community across butterfly life stages. Panels show constrained analysis of principal coordinates (CAP) for larvae, pupae and adults of each species based on the composition and relative abundance of bacterial OTUs after applying a 5% abundance cut-off. Axis labels indicate first two linear discriminants (LD1 and LD2) that best explain the classification of samples in different groups (larvae, pupae and adults; see Material and methods). Ellipses represent 95% confidence intervals. Red asterisks denote significant variation across larvae and adults, and black asterisks denote significant variation in bacterial communities across larvae, pupae and adults (electronic supplementary material, table S1D, MANOVA, *p* < 0.05). For panels (vi–ix), the first linear discriminant explained all of the variation, and is thus represented as a single axis (see electronic supplementary material, table S1D).

We analysed variation in bacterial community structure across developmental stages of butterfly species (figure 2*a,b*; electronic supplementary material, table S1A–S1D), focusing on the top 5 OTUs (figure 2*a*) and OTUs with 5% abundance cut-off (figure 2*b*). Our dataset included nine species of butterflies with samples from at least two developmental stages. Of these, for eight species we compared larvae and adults; for six species we compared larvae, pupae and adults; and for one species we compared larvae and pupae (table 1; electronic supplementary material, table S1A and S1B). We expected that bacterial communities of larvae and adults should vary substantially owing to the dramatic difference in their diets. However, we found that only one (of eight) species had significantly different larval and adult communities (*p* = 0.04, *R*^2^ = 0.53, 5% abundance cut-off, electronic supplementary material, table S1A), and two other species showed marginally significant differences (*p* = 0.05, *R*^2^ = 0.22 and *p* = 0.07, *R*^2^ = 0.28; 5% abundance cut-off*,* electronic supplementary material, table S1A; results were similar when considering top 5 OTUs). For the remaining five butterfly hosts, life stage accounted for 6–32% of the variation in bacterial communities, but this impact was not significant (electronic supplementary material, table S1A). These results suggest that dietary transitions during development may not have a strong impact on bacterial communities of most butterfly hosts.

When we included the non-feeding pupal stage in this comparison, we found that four out of seven butterfly species showed significant stage-specific variation in bacterial communities (figure 2*a,b*; electronic supplementary material, table S1B). We obtained similar results using all other OTU filtering cut-offs (electronic supplementary material, tables S1A and S1B). We also found similar levels of bacterial community richness and evenness across different life stages (electronic supplementary material, figure S5). Thus, overall we found weak and variable impact of developmental differences in butterfly-associated bacterial communities. An extreme example is the butterfly *L. plinius*, where the dominant bacterial community was almost identical across larvae and adults (figure 2*a*(vi)).

We tested whether our results are confounded by variation across sequencing runs and variation across individual hosts. We separately analysed bacterial community dynamics across host development for samples processed in each sequencing run, and found that the results obtained from each run are similar to our finding with pooled samples (electronic supplementary material, table S1C). Next, we examined variation in the abundance of dominant bacterial OTUs across replicate host samples. We found substantial individual variation in developmental stages of some host species (electronic supplementary material, figure S6 and table S2). To test whether greater individual-level variation may have masked the impact of development in some butterfly species, we calculated the coefficient of variation (CV) for each of the top 5 bacterial OTUs for each developmental stage of a host species (electronic supplementary material, figure S7). However, CV values were not significantly different across butterfly hosts that showed a significant versus non-significant impact of developmental stage on bacterial communities. Hence, we cannot attribute the lack of a developmental signal on bacterial community composition to greater inter-individual variability in some hosts. Together, our results indicate that dietary and developmental switches in butterflies have variable but generally weak impacts on their bacterial communities However, because we used whole insect bodies for microbiome analysis, we acknowledge that these results do not account for potential location-specific variation in bacterial abundance across development [50].

### 3.3. Host identity significantly impacts bacterial community structure of butterflies

Next, we tested the impact of host identity (species and family) on bacterial community structure. We analysed bacterial communities of larvae, pupae, adults and all stages (pooled) across host species and families, using constrained as well as unconstrained ordination analysis. For ordination, we used the 5% OTU abundance cut-off.

**Figure 3.**
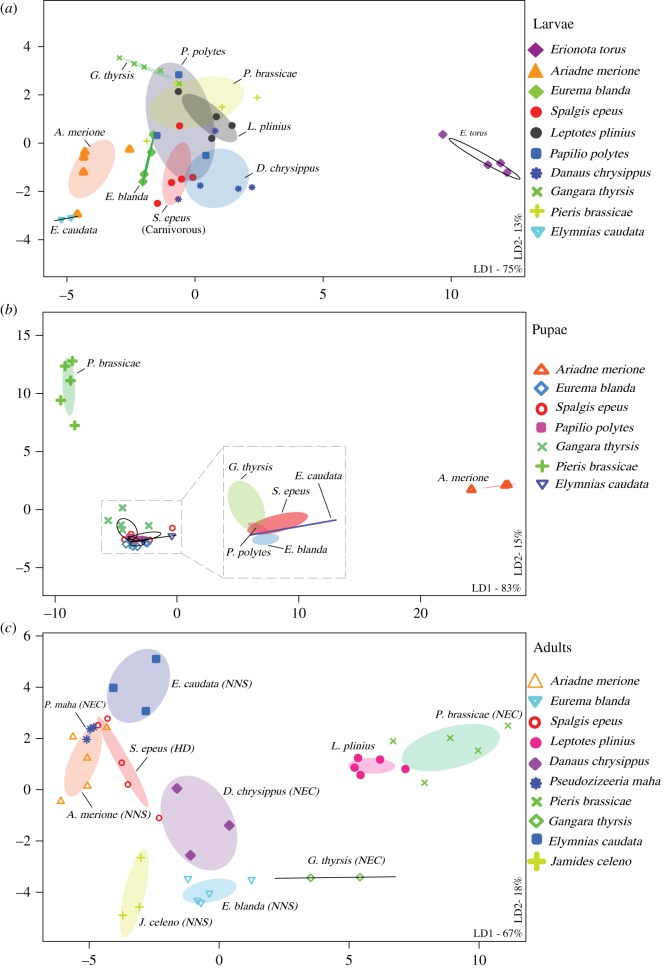
Variation in bacterial communities of developmental stages of different butterfly host species. Panels show constrained analysis of principal coordinates (CAP) for larvae (*a*), pupae (*b*) and adults (*c*) based on the composition and relative abundance of bacterial OTUs after applying a 5% abundance cut-off. Axis labels indicate the proportion of between-group variance (%) explained by the first two linear discriminants (LD1 and LD2). Ellipses represent 95% confidence intervals. For each panel, we observe a significant effect of host species (*p* < 0.05, multivariate ANOVA). Carnivorous *S. epeus* larvae (*a*) and the adult dietary resource (table 1) of each butterfly species are marked (*c*). In panel (*b*), the plot area marked with a square is expanded to clearly show ellipses. In panel (*a*), the impact of host species remained significant even after removing the potential outlier *E. torus* larvae (electronic supplementary material, figure S12; MANOVA, *p* < 0.05).

Within each developmental stage, we found significant variation in bacterial communities across butterfly species (MANOVA, *p* < 0.05; figure 3), with the first two linear discriminants (LD1 + LD2) capturing greater than 80% between-group variation (electronic supplementary material, table S3A). The total between-group variation explained (LD1 + LD2) was highest for pupae (98%) and lower for larvae (88%) and adults (85%) (electronic supplementary material, table S3A). However, when using other rare-OTU filters, we observed that total LD explained was greater for adults than larvae (electronic supplementary material, table S3A). Similarly, a PERMANOVA analysis showed that host species identity explained slightly more variation in the microbiomes of adults (approx. 8% more than larvae; electronic supplementary material, table S4A). Although it is unclear whether this difference is biologically meaningful, the pattern is opposite to our expectation of stronger host-specificity in larvae rather than adults. Overall, the impact of host species was much weaker if we pooled individuals across developmental stages (electronic supplementary material, tables S3A and S4, figure S8), indicating that host effects vary across developmental stages.

**Figure 4.**
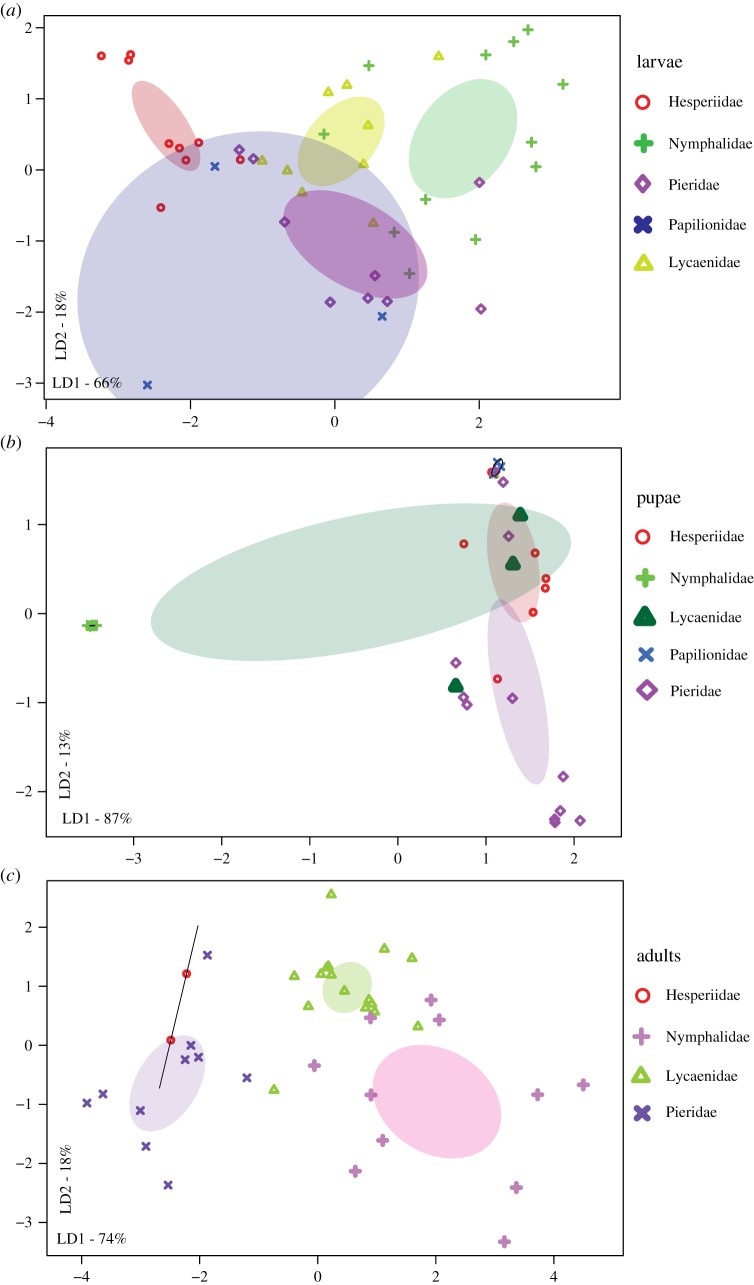
Variation in bacterial communities across butterfly host families: (*a*) larvae, (*b*) pupae and (*c*) adults. Panels show constrained analysis of principal coordinates (CAP) for OTUs from larvae, pupae and adults after applying a 5% abundance cut-off. We pooled all individuals belonging to a butterfly taxonomic family, regardless of their species. Axis labels indicate the proportion of between-group variance (%) explained by the first two linear discriminants (LD1 and LD2). Ellipses represent 95% confidence intervals. For each panel, we observe a significant effect of host family (*p* < 0.05, multivariate ANOVA).

As with host species, host taxonomic family also significantly affected bacterial community structure (electronic supplementary material, tables S3A and S4; figure 4). For larvae (figure 4*a*), family Papilionidae showed substantial overlaps with other families based on 95% confidence ellipses. However, family Papilionidae was represented by only one species (*P. polytes*). When we performed the same analysis without family Papilionidae, we observed distinct clusters of larval families with some overlap between family Lycaeniade and Pieridae (electronic supplementary material, figure S9). Unlike the patterns observed for host species, the total variation explained by host family was not different for larvae and adults (electronic supplementary material, table S4). Comparing the relative impact of host species and family on community composition in each developmental stage, we found that host species typically explained greater total variation (approx. 30% more for larvae and adults, and approx. 10% more for pupae; electronic supplementary material, table S4). This suggests that bacterial communities are more strongly structured by host species rather than host family. When we repeated the analyses separately with samples for each sequencing run (electronic supplementary material, table S4B), different OTU cut-offs (electronic supplementary material, table S4C) and using rarefaction (electronic supplementary material, table S4D), we found similar results. The overall patterns were generally consistent even if we removed the potential endosymbiont *Wolbachia* from our analysis (electronic supplementary material, figures S10 and S11, table S3B); although removing *Wolbachia* decreased the variation explained by total LD (electronic supplementary material, table S3B) and reduced the impact of host family on larval bacterial communities (MANOVA, *p* = 0.06).

Next, we performed unconstrained principle coordinates analysis to test whether host species and families cluster separately without *a priori* grouping (electronic supplementary material, figure S13 and S14). As with constrained ordination, we plotted PCoA ordination plots using a 5% abundance cut-off (electronic supplementary material, figures S13A and S14A). We also used the unfiltered OTU set (most permissive filter) and core OTU set (stringent filter) to visualize the spread of individual samples in the ordination plot (electronic supplementary material methods, figures S13B,C and S14B,C). Similar to results for the constrained ordination analysis, we found significant variation in bacterial communities across host species and families, suggesting that different butterflies harbour phylogenetically distinct bacterial communities (electronic supplementary material, table S5). The unconstrained analysis also showed that host species identity explained a greater fraction of the total variation in bacterial communities of adults, compared to communities of larvae (electronic supplementary material, table S5).

### 3.4. Diet affects bacterial communities across butterfly host species

**Figure 5.**
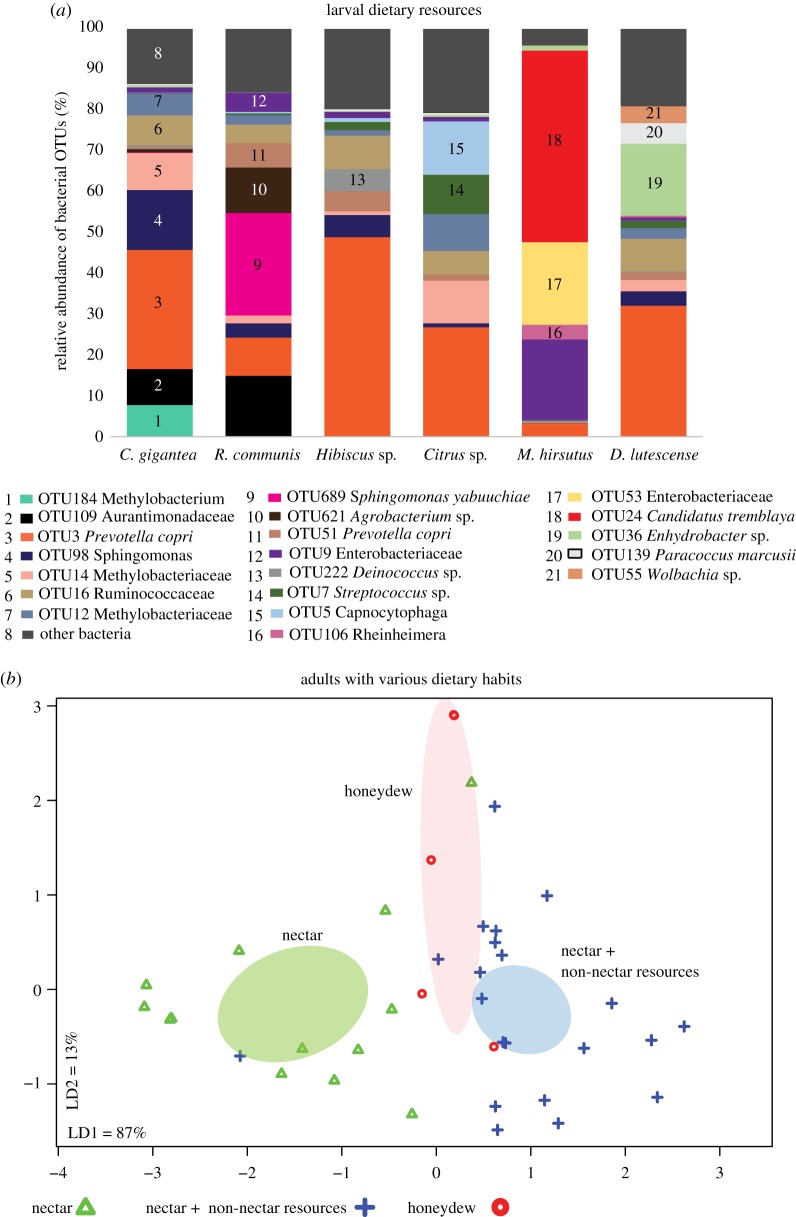
Variation in bacterial community composition across butterfly dietary resources. (*a*) Stacked bar plots show the average relative abundance of the top 5 dominant bacterial OTUs across all larval dietary resources. (*b*) Variation in bacterial communities of adults with different dietary habits (table 1). Panel shows constrained analysis of principal coordinates (CAP) of OTUs for adults from different dietary guilds after applying a 5% abundance cut-off. Axis labels indicate the proportion of between-group variance (%) explained by the first two linear discriminants (LD1 and LD2). Ellipses represent 95% confidence intervals. We observe a significant effect of dietary resources on adult bacterial communities (*p* < 0.05, multivariate ANOVA).

**Figure 6.**
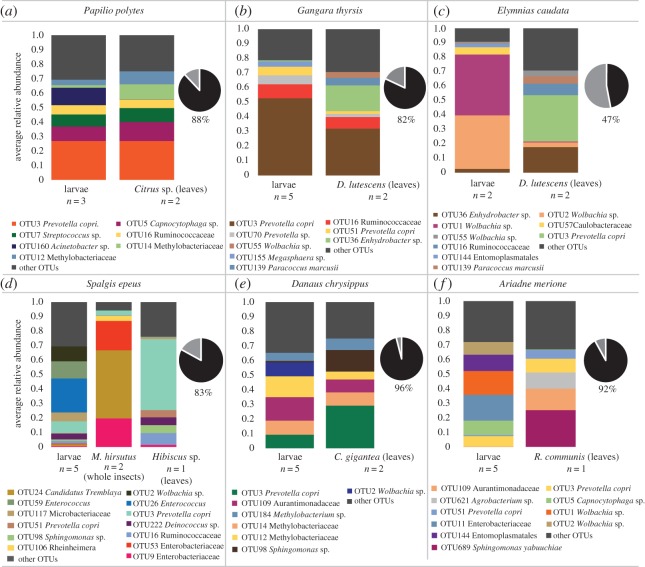
(*a*–*f*) Bacterial community composition of larvae and their diets. Stacked bar plots represent the average relative abundance of the five most abundant bacterial OTUs. Each bar represents a larval stage or a dietary resource; *n* = number of replicates sampled. Each panel shows data for a different butterfly species. Variation in bacterial community composition was tested using permutational multivariate ANOVA (PERMANOVA, 1000 permutations). For *G. thyrsis*, *p* < 0.05; for all other comparisons, *p* > 0.05. Pie charts in each panel represent the proportion (%) of dietary bacteria (OTUs) found in larvae (black slice) at the 5% relative abundance cut-off.

The strong host-specificity observed in bacterial communities could arise through neutral mechanisms (e.g. distinct input communities due to dietary differences), via host-imposed selection (e.g. due to dietary or physiological differences) or a combination of both processes. To determine the importance of dietary differences (through neutral or selective mechanisms of community assembly), we compared bacterial communities associated with larval dietary sources (table 1 and figure 5*a*). We found that bacterial communities varied significantly across dietary resources (table 1; PERMANOVA, d.f. = 3, *R*^2^ = 0.73105, *p* = 0.03, 5% OTU abundance cut-off). These results indicate a potential role for larval diets in driving host-specific bacterial communities. Interestingly, in five of six species, approximately 80% of the OTUs found in dietary resources were also found in the dominant bacterial communities of larvae (figure 6; electronic supplementary material, table S6). Hence, it is not surprising that the bacterial community structure of larvae and their diets did not vary significantly (figure 6; PERMANOVA, *p* > 0.05) except in *G. thyrsis* (PERMANOVA, *p* = 0.047). Notably, the carnivorous larvae of *S. epeus* shared approximately 75% of their bacterial community with that of their insect prey *M. hirsutus* (mealybugs) and approximately 50% of the community with the mealybugs' host plant *Hibiscus* (figure 6*d*; electronic supplementary material, table S6). Overall, our results suggest that the bacterial communities of butterfly larvae are largely shaped by their diet.

For wild-caught adults, we had no information on specific dietary resources. However, we compared bacterial communities across host species known to vary in their dietary habits (table 1 and figure 5*b*). Our adult butterfly species included three broad dietary types, with species that feed on (i) nectar only, (ii) nectar + non-nectar resources, and (iii) honeydew (table 1). A CAPdiscrim analysis showed significant variation across these groups (LD1 + LD2 = 100%, classification success = 79%, MANOVA *p* = 0.007; 5% abundance cut-off, figure 5*b*), as did a PERMANOVA analysis (d.f. = 2, *R*^2^ = 0.092, *p *= 0.0054, 5% abundance cut-off). In fact, we observed significant host specificity within the dietary guild with multiple host species (PERMANOVA for species with NNS diet in table 1; d.f. = 4, *R*^2^ = 0.39, *p* = 0.0002, 5% abundance cut-off), suggesting that each of these species may use distinct non-nectar resources. Note that we removed *Wolbachia* OTUs for this analysis, because they may represent non-gut associated endosymbionts. Together, our results suggest that dietary variation may explain the observed host-specificity in butterfly-associated bacterial communities. However, whether the impact of diet reflects neutral (passive acquisition) or host-mediated selective processes remains unclear.

## 4. Discussion

Across animals, a large body of work shows that diet plays a major role in shaping host-associated microbial communities [51–56]. We demonstrate that although this pattern holds across butterfly species, the dramatic dietary shifts across developmental stages of a given host have a very weak role in shaping bacterial community structure. This is surprising because differences in diet quality and physiological variation across metamorphosis should generate strong selection to maintain distinct sets of beneficial microbes in each life stage. For instance, the larval diet (mostly leaves) can be difficult to digest as it is typically enriched in cellulose, hemicellulose, lignin, pectin [57] and several toxic plant defence compounds [58]; and is often nitrogen limited [7] . Conversely, the adult diet (primarily nectar for species in this study) is typically composed of sucrose, glucose, hexose, fructose and amino acids [59,60], with fewer or no toxins because plants attract pollinators with the nectar reward. In spite of this stark variation in diet, we did not find significant changes in bacterial communities across larvae and adults for most butterflies.

The overall lack of developmental signal could arise via two mechanisms. First, larvae and adults of a given host species could independently acquire similar bacteria from their respective diets. Second, a large proportion of the larval bacterial community may be maintained across metamorphosis, causing significant overlap in bacterial communities of larvae and adults. The first scenario is less likely, because previous reports show that leaves and nectar of different plant species harbour distinct bacterial communities [61–64]; thus, it is unlikely that larvae and adults would take up similar bacteria from their respective diets. However, we found partial support for the second hypothesis: in some butterflies (three of seven species with pupal sampling), we found that pupae had similar bacterial composition and richness as that of the larvae and adults (figure 2; electronic supplementary material, figure S3). This is in contrast to previous reports with other insects, where pupae seem to harbour fewer OTUs relative to larvae and adults [29,31,34,40,65]. In fact, we found cases where the diversity and abundance of bacterial OTUs increased during the pupal stage (figure 2; electronic supplementary material, figure S3). For instance, in *P. polytes*, the average relative abundance of OTU7 (*Streptococcus sp.*) increased roughly 200% from larvae to pupae, and in *P. brassicae* the average relative abundance of OTU25 and OTU56 (family Enterobacteriaceae) increased by 45% and 85%, respectively, from larvae to pupae. Similarly, in *S. epeus* and *G. thyrsis*, OTU richness in pupae was higher than in larvae and adults. However, because our data represent the relative abundance of bacteria, these perceived changes in bacterial enrichment and diversity can also result from a change in the relative abundance of other community members. An interesting avenue for further work is to identify where and how bacteria are maintained or enriched in pupae, and whether the enrichment is beneficial for the host (e.g. using quantitative PCR in conjunction with manipulative experiments). This is especially relevant for understanding instances where we observed significant changes in bacterial communities across developmental stages. In the moth *Galleria mellonella*, symbiotic bacteria interact with the host immune system to ensure the transmission of gut microbes through metamorphosis [66]. Such a mechanism may also allow similar bacterial communities to be maintained across larvae and adults of some of our focal butterflies.

Unlike developmental stage, we found that host species and taxonomic family strongly impacted bacterial community structure. An earlier analysis of bacterial communities across 39 insect species from 28 families found similar results, showing that closely related insect taxa have more similar bacterial communities [67]. Additionally, a recent report on microbiomes of adult butterflies also suggests that bacterial communities vary significantly across butterfly species [48]. We expected a strong impact of host species identity across larvae of different species, because bacterial communities vary significantly across plant species [49,61,62], and should be reflected in distinct larval microbiomes even in the absence of selection. Specifically, larvae of our focal butterfly species feed on distinct host plants with distinct microbiomes (figures 5 and 6). On the other hand, adults of many butterfly species are generalists [68,69]. Therefore, we expected specific associations between larvae and bacterial partners but relatively weaker associations for adults. To the contrary, we found that the impact of host species on bacterial communities of larvae and adults was slightly greater in adults (electronic supplementary material, table S4A and S4C). For larvae, we found substantial overlap with the bacterial communities associated with larval diet, indicating that larval bacterial communities are largely shaped by passive acquisition of bacteria from dietary resources. This pattern is consistent with weak selection for host–bacterial associations in butterfly larvae, as suggested by a recent analysis of Lepidopteran (mostly moth) larvae [49]. The stronger signal of host-specificity in adults could arise if different species specialize on different nectar or non-nectar resources, acquiring distinct sets of bacteria [63,64]. Indeed, we observed that broad dietary groups explained a large proportion of variation in bacterial community structure in adults. Another possibility is that adults impose a stronger physiological filter on bacterial communities than larvae. This hypothesis is supported by a recent study [48] showing that bacterial communities of neotropical butterfly adults are highly dissimilar to that of their diet. In addition to species identity, taxonomic family also significantly impacted bacterial community structure across butterflies. This effect can arise due to several factors such as variation in dietary guilds, divergent, family-specific host physiology and immune system, both of which may select for distinct sets of bacteria. Further work is necessary to test these hypotheses and determine the relative role of neutral and selective processes in shaping butterfly-associated bacterial communities.

With respect to bacterial taxonomy, our findings are generally congruent with previous work on insect-associated bacterial communities. The most abundant and frequent OTUs in butterflies are also common members of other insect-associated microbiomes. For instance, of the 12 frequent bacterial OTUs found across butterflies, families Methylobacteriaceae, Enterobacteriaceae and genus *Wolbachia* together represented seven OTUs. These bacteria also dominate bacterial communities associated with insects from multiple orders [24], including Lycaenid butterfly larvae [28]. Similarly, the phyla Proteobacteria, Firmicutes, Bacteroidetes and Actinobacteria accounted for approximately 94% of the total bacterial abundance in butterflies; these phyla are again commonly observed in multiple insects [23,24,67] including the butterfly *Heliconius erato* [40] and butterfly larvae from family Lycaenidae [28]. Such widespread insect-bacterial co-occurrence may represent evolved functional relationships, allowing the bacteria to easily colonize and proliferate within a wide range of insects including butterflies. On the other hand, they may simply reflect the fact that these bacteria are commonly found in the phyllosphere and soil [70,71] that serve as ecological or dietary niches for many insects, including butterflies. Further work is necessary to distinguish between these hypotheses.

A large body of work has analysed microbial communities associated with insects [18,23,24,72–74], but very few studies have investigated butterflies. There are about 19 000 species of butterflies worldwide [75] that exhibit large diversity in their dietary habits, ecological niches and life history. They are important pollinators and herbivores, while some are pests in the larval stages [76–78]. Butterflies are also used as model systems across several disciplines of biology such as genetics, behavioural ecology, developmental biology and evolutionary biology [79]. Despite being such an ecologically important insect group and a widely used model system, very little is known about the bacterial communities associated with butterflies. Our study is one of the first investigations of bacterial communities harboured by a diverse set of wild-caught butterfly species, across developmental stages and larval diets. Surprisingly, we find that despite the large dietary switch, butterfly larvae and adults have fairly similar bacterial communities, with weak evidence of specific co-evolved associations. Our work highlights the importance of comparative analyses across multiple species within an insect group, because focusing on any one butterfly species might have led to different conclusions. Further studies can build upon our results by including butterfly species with more diverse diets and better resolution across developmental stages; and by conducting manipulative experiments to test whether butterfly–bacterial associations are mutualistic, commensal or parasitic.

## Supplementary Material

Supplementary Methods

## Supplementary Material

Supplementary Figures

## Supplementary Material

Supplementary Tables

## Data accessibility

Raw Fastq files generated through illumina MiSeq amplicon sequencing are uploaded in the European Nucleotide Archive (EMBL EBI) database (www.ebi.ac.uk/) under accession number PRJEB21255. Raw Fastq files will be publicly available post publication. All the supporting information such as sample information, R codes and raw OTU tables are uploaded on Dryad Digital Repository at: https://doi.org/10.5061/dryad.b01k5 [80].
