# Supplementary material for: Dietary and developmental shifts in butterfly-associated bacterial communities
Source: R Soc Open Sci. 2018 May 30;5(5):171559. doi: 10.1098/rsos.171559 (PMC5990769; doi:10.1098/rsos.171559)
Supplement: Supplementary Methods [file rsos171559supp1.pdf]

DIETARY AND DEVELOPMENTAL SHIFTS IN BUTTERFLY-ASSOCIATED BACTERIAL COMMUNITIES

Kruttika Phalnikar*, Krushnamegh Kunte and Deepa Agashe*

National Centre for Biological Sciences (NCBS), GKVK Campus, Bellary Road, Bangalore, India 560065

*Corresponding authors:

kruttikap@ncbs.res.in

dagashe@ncbs.res.in

Supplementary Methods

Testing for bacterial contamination:

As reported by a previous study [1] DNA extraction kits can introduce bacterial contaminants. To test for contamination, we carried out a mock DNA extraction using all the kit reagents (negative control). As a positive control, we used larval DNA of *A. merione* and *D. chrysippus*. We prepared 16S rRNA libraries and quantified the amount of DNA after each PCR purification step. We used Qubit (Qubit Fluorometric Quantitation, Thermo Scientific), a highly sensitive method for DNA quantification. PCR with larval samples yielded ~700 ng and ~3000 ng of total DNA after the first and second round of PCR purification respectively. In the negative control, we could not detect any DNA using Qubit after either round of PCR purification. Thus, it is unlikely that our DNA extraction kits introduced large amounts of contaminating 16S reads in our dataset.

Analysing bacterial community structure:

We quality filtered and analysed our data in QIIME (Quantitative Insights Into Microbial Ecology) version 1.9.1 [2]. We used 97% sequence similarity to define bacterial OTUs. We avoided summarizing bacterial OTUs in different taxonomic groups since each group can contain several OTUs with very different relative abundances, leading to confusion while statistically comparing relative abundance across host samples. We used the following commands in QIIME to obtain the final bacterial OTU table. For most of the QIIME commands we used the default parameters. The parameter specifications (if changed from the default value) are mentioned in parentheses.

- Joining forward and reverse paired ends of the reads- *multiple_join_paired_ends.py*
- Filtering low quality reads - *multiple_split_libraries_fastq.py* (q score >19)
- Identifying and filtering chimeric sequences with Usearch61 - *identify_chimeric_seqs.py* and *filter_fasta.py*
- Pick OTUs - *pick_open_reference_otus.py*

- Remove Chloroplast and Mitochondria from the OTU table - *filter_taxa_from_otu_table.py*

After obtaining the number of reads for each bacterial OTU in each sample, we obtained relative abundance for each OTU in each sample. We further implemented 5 different filtering cut-offs to remove rare OTUs from our analysis that may not contribute significantly to the bacterial community or may have arisen due to sequencing errors. These cut-offs were as follows:

(A) <u>Minimum OTU abundance of 5% in at least 1 sample</u>

As an alternative to the set of dominant bacteria described above, we selected OTUs that contributed ≥ 5% relative abundance in at least 1 sample within the entire dataset. Even if all but one sample had ≥5% relative abundance for an OTU, it was thus retained in the trimmed dataset. As a result, we chose a large fraction of the full 16S-derived community for comparative analysis, while removing most of the rare members

(B) <u>Selecting top 5 abundant OTUs</u>

To determine the most dominant set of OTUs, we selected the 5 most abundant OTUs (from 5% cut-off OTU set as described above) for each developmental stage for each butterfly species. This small subset of bacterial OTUs also allowed better visualization of the dynamics of dominant community members across butterfly development. To select the top 5 bacterial OTUs, we first calculated the mean relative abundance of each bacterial OTU across all replicates of a developmental stage of a host species. For each stage, we sorted the mean relative abundance by rank and created a combined dataset of the 5 most abundant OTUs across all stages of a host species. For instance, if OTU X was one of the top 5 OTUs in larvae but not in adults, it was still selected as a part of the top 5 bacterial OTU dataset for that butterfly host, with the appropriate mean relative abundance included for each developmental stage. This is exemplified in the tables below. For some species the top 5 OTUs (T5) were similar across larvae, pupae and adults (Table A, Species 1) whereas for some butterflies they differed across stages (Table B, Species 2). As a result, for each butterfly species, we obtained a slightly different number OTUs in a top 5 set e.g. 5 OTUs for species 1 and 7 OTUs for species 2.

(A) butterfly 1	Larvae	Pupae	Adults
Bacterial OTU a	T5	T5	T5
Bacterial OTU b	T5	T5	T5
Bacterial OTU c	T5	T5	T5
Bacterial OTU d	T5	T5	T5
Bacterial OTU e	T5	T5	T5

(B) butterfly 2	Larvae	Pupae	Adults
Bacterial OTU a	T5	T5	T5
Bacterial OTU b	T5	-	-
Bacterial OTU c	T5	T5	T5
Bacterial OTU d	T5	T5	-
Bacterial OTU e	T5	-	T5
Bacterial OTU f	-	T5	T5
Bacterial OTU g	-	T5	T5

We applied a similar method of OTU selection for other analyses such as comparing bacterial communities of larvae vs. larval diet.

(C) Minimum OTU abundance of 0.005% across the entire dataset

Next, we excluded rare OTUs based on their abundance across all samples in our original dataset (instead of allowing high abundance in at least one sample, as explained above). We calculated the ratio of total reads contributed by the OTU to the total number of reads obtained across the entire dataset (not per sample), and removed OTUs with a total relative abundance less than 0.005%. We selected this cut-off based on a previous study that reviewed sequencing data generated across several sequencing platforms and found that this cut-off helps eliminate OTUs that may arise due to Illumina sequencing errors [3].

(D) Minimum 20 reads per OTU

We filtered out only those OTUs with low sequencing coverage (rather than filtering by relative abundance). For each sample, we removed OTUs that had less than 20 reads. We expected this filtering to remove any spurious OTUs that could have arisen due to PCR or sequencing errors. Previous studies have removed single or doubletons [4–6] and occasionally have also applied the cut-off of minimum 10 reads [7–9]. However, in order make our analysis stringent we applied a cut-off of minimum 20 reads per OTU per sample.

(E) Selecting Core OTUs

In addition to various abundance based cut-offs (A-D), we next used a frequency based cut-off to extract bacterial OTUs that occur in >80% of the samples – the “Core OTU" set – using the “compute_core_microbiome.py” function in Qiime 1.9.1. With this set of OTUs, bacterial communities of samples from run 1 and run 2 showed 100% overlap, as expected.

After applying each cut-off independently, we recalculated the relative abundance of OTUs for subsequent downstream analysis. The OTU filtering was done in Microsoft Excel and using customized algorithms written in R [10].

Statistical analysis

We used R [R Core Team 2013] for all statistical analyses. To test the variation in bacterial community structure across different treatment groups (e.g. species, family), we performed Permutational Multivariate Analysis of Variance (PERMANOVA) using the Adonis function in the R package Vegan[11]. To visualise and quantify the impact of treatment groups on bacterial community composition we separately used constrained or unconstrained ordination.

For constrained ordination, we used Canonical Analysis of Principal Coordinates based on Discriminant Analysis (CAPdiscrim) in the R package “BiodiversityR” [12]. This function, in association with the R package “Vegan”, converts the abundance matrix of all OTUs into a distance matrix using Bray-Curtis distances. We also used this package to perform a linear discriminant analysis (LDA) on the principal co-ordinate (PCo) values generated from the distance matrix, to find the impact of treatment groups on bacterial community structure. LD plots show the clustering of data points based on the linear discriminants with highest contribution to between-group variation. We added an ellipse showing 95%

confidence intervals (using standard error) using the function “Ordiellipse” in R package “Vegan” [13] in order to visually differentiate the clusters. To statistically test the impact of different treatment groups on variation in bacterial communities, we used MANOVA.

In addition to CAPdiscrim, we carried out an unconstrained analysis of principal co-ordinates (PCoA) to test the separation between treatment groups and variation across replicates, based on the phylogenetic distance between bacterial OTUs. For this analysis, we calculated beta diversity of bacterial OTUs using weighted unifrac distance followed by generation of principle coordinates that were used for plotting ordination. For this analysis we used Qiime 1.9.1 [14]. We calculated beta diversity using function *beta_diversity.py* and plotted PCoA using *principal_coordinates.py* and *make_2d_plots.py* in Qiime 1.

CAPdiscrim is a constrained ordination analysis method, where groups are defined *a priori* and LDA maximises between group variation. For our analysis, we report the 3 main outputs of CAPdiscrim, a) classification success, b) proportion of trace (LD1, LD2) and c) p value of MANOVA. Percent classification success suggests how well groups can be distinguished, whereas proportion of trace (LD1 and LD2) represents the linear discriminants that best contribute to the classification success and explain maximum between-group variance. On the other hand, PCoA is an unconstrained analysis where samples are not categorized into groups. PCoA creates an ordination that best explains the variation across the entire dataset.

REFERENCES

1. Salter, S. J., Cox, M. J., Turek, E. M. & Calus, S. T. In press. Reagent and laboratory contamination can critically impact sequence-based microbiome analyses. *bmcbiol.biomedcentral.com*

2. Caporaso, J. G. et al. 2010 QIIME allows analysis of high-throughput community sequencing data. *Nat. Methods* **7**, 335–336. (doi:10.1038/nmeth.f.303)

3. Bokulich, N. A., Subramanian, S., Faith, J. J., Gevers, D., Gordon, J. I., Knight, R., Mills, D. A. & Caporaso, J. G. 2013 Quality-filtering vastly improves diversity estimates from Illumina amplicon sequencing. *Nat. Methods* **10**, 57–59. (doi:10.1038/nmeth.2276)

4. Allen, H. K., Bayles, D. O., Looft, T., Trachsel, J., Bass, B. E., Alt, D. P., Bearson, S. M. D., Nicholson, T. & Casey, T. A. 2016 Pipeline for amplifying and analyzing amplicons of the V1-V3 region of the 16S rRNA gene. *BMC Res Notes* **9**, 380. (doi:10.1186/s13104-016-2172-6)

5. Gibson, J., Shokralla, S., Porter, T. M., King, I., van Konynenburg, S., Janzen, D. H., Hallwachs, W. & Hajibabaei, M. 2014 Simultaneous assessment of the macrobiome and microbiome in a bulk sample of tropical arthropods through DNA metasystematics. *Proc. Natl. Acad. Sci. USA* **111**, 8007–8012. (doi:10.1073/pnas.1406468111)

6. Majaneva, M., Hyytiäinen, K., Varvio, S. L., Nagai, S. & Blomster, J. 2015 Bioinformatic amplicon read processing strategies strongly affect eukaryotic diversity and the taxonomic composition of communities. *PLoS One* **10**, e0130035. (doi:10.1371/journal.pone.0130035)

7. Lau, J. T., Whelan, F. J., Herath, I., Lee, C. H., Collins, S. M., Bercik, P. & Surette, M. G. 2016 Capturing the diversity of the human gut microbiota through culture-enriched molecular profiling. *Genome Med.* **8**, 72. (doi:10.1186/s13073-016-0327-7)

8. Franzén, O., Hu, J., Bao, X., Itzkowitz, S. H., Peter, I. & Bashir, A. 2015 Improved OTU-picking using long-read 16S rRNA gene amplicon sequencing and generic hierarchical clustering. *Microbiome* **3**, 43. (doi:10.1186/s40168-015-0105-6)

9. Werner, J. J., Zhou, D., Caporaso, J. G., Knight, R. & Angenent, L. T. 2012 Comparison of Illumina paired-end and single-direction sequencing for microbial 16S rRNA gene amplicon surveys. *ISME J.* **6**, 1273–1276. (doi:10.1038/ismej.2011.186)

10. Team, R. 2013 R development core team. *RA Lang Environ Stat Comput*

11. Oksanen, J., Kindt, R., Legendre, P., O'Hara, B. & et al. 2007 The vegan package. *Community ecology …*

12. Kindt, R. & Kindt, M. R. 2007 The BiodiversityR Package.

13. Oksanen, J., Blanchet, F. G., Kindt, R. & et al. 2013 Package' ' 'vegan. *Community ecology …*

14. Caporaso, J. G., Kuczynski, J., Stombaugh, J. & Bittinger, K. In press. QIIME allows analysis of high-throughput community sequencing data. *nature.com*
