# Supplementary material for: Dietary and developmental shifts in butterfly-associated bacterial communities
Source: R Soc Open Sci. 2018 May 30;5(5):171559. doi: 10.1098/rsos.171559 (PMC5990769; doi:10.1098/rsos.171559)
Supplement: Supplementary Figures [file rsos171559supp2.pdf]

DIETARY AND DEVELOPMENTAL SHIFTS IN BUTTERFLY-ASSOCIATED BACTERIAL COMMUNITIES

Kruttika Phalnikar*, Krushnamegh Kunte and Deepa Agashe*

National Centre for Biological Sciences (NCBS), GKVK Campus, Bellary Road, Bangalore, India 560065

*Corresponding authors:

kruttikap@ncbs.res.in

dagashe@ncbs.res.in

Supplementary Figures

Figure S1: Proportion of mitochondria and chloroplast in each sample. Barplots show the relative proportion of sequences categorized as mitochondria and chloroplast for each sample. The average proportion of mitochondria and chloroplast together was ~13% across all butterfly samples ranging from 0% to 98%

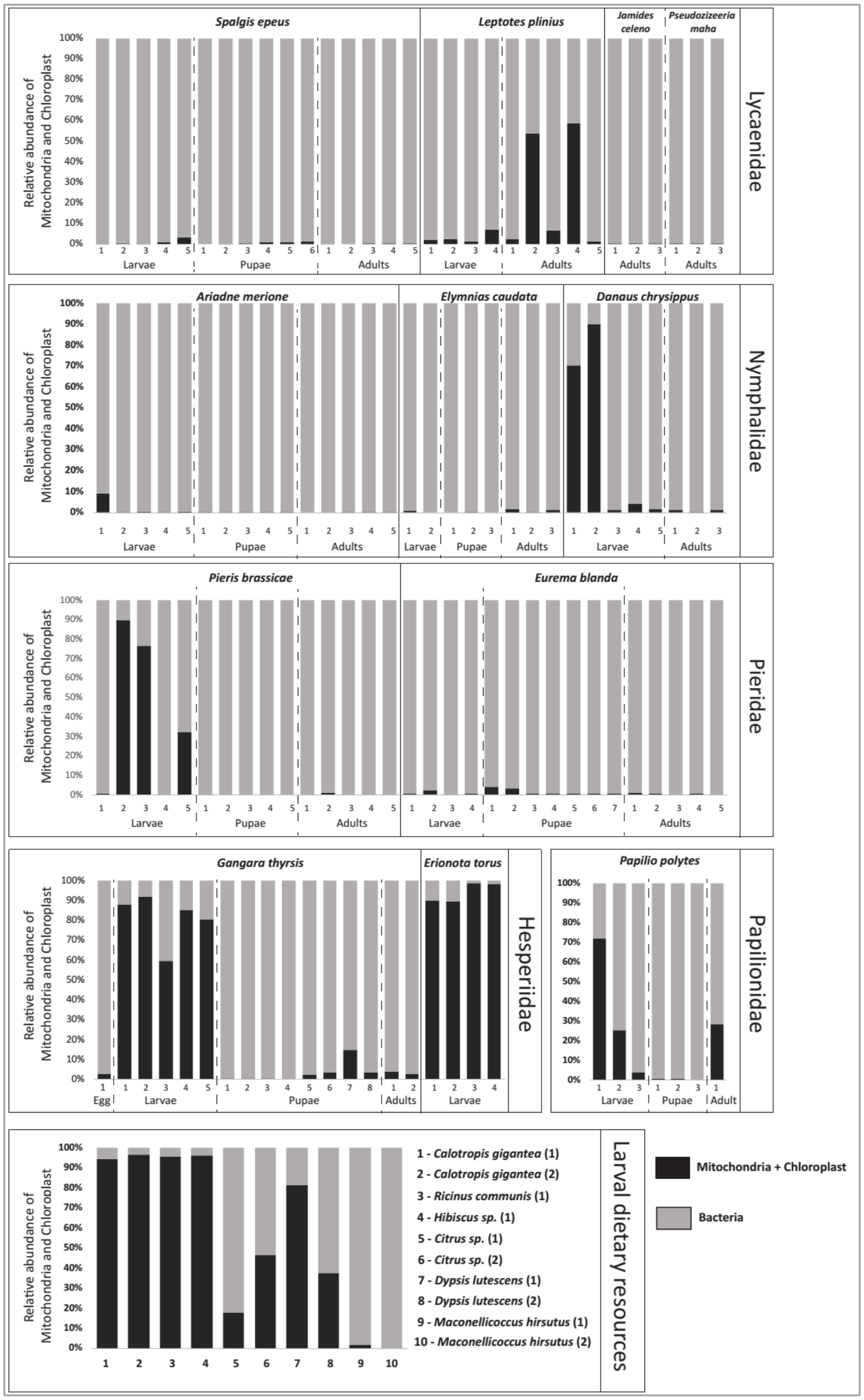

Figure S2: Variation in sequencing depth, bacterial OTUs per sample, and bacterial composition across two Illumina MiSeq sequencing runs. (A) Average number of reads obtained per sample calculated without applying any rare OTU filtering cut-off. **(B-E)** Average number of bacterial OTUs present in each sample across two sequencing runs after applying different rare OTU filtering cut-offs (see Methods and SI Methods). Bacterial OTU richness becomes comparable across run 1 and run 2 after filtering the bacterial community with 5% abundance cut-off. Error bars represent standard deviation. **(F)** Compositional variation in Run 1 and Run 2 after applying the >5% abundance cut-off. Total 98 OTUs were obtained after applying the 5% abundance cut-off, of which, 84 were shared across both runs. **(G-H)** Variation in unfiltered bacterial communities across run 1 and run 2 reduces after filtering the community to select for core OTUs (present in minimum 80% of the samples). Panels G and H show the principle coordinates analysis of unfiltered bacterial OTUs (G) and core bacterial OTUs (H) based on a phylogenetic distance metric (Weighted Unifrac).

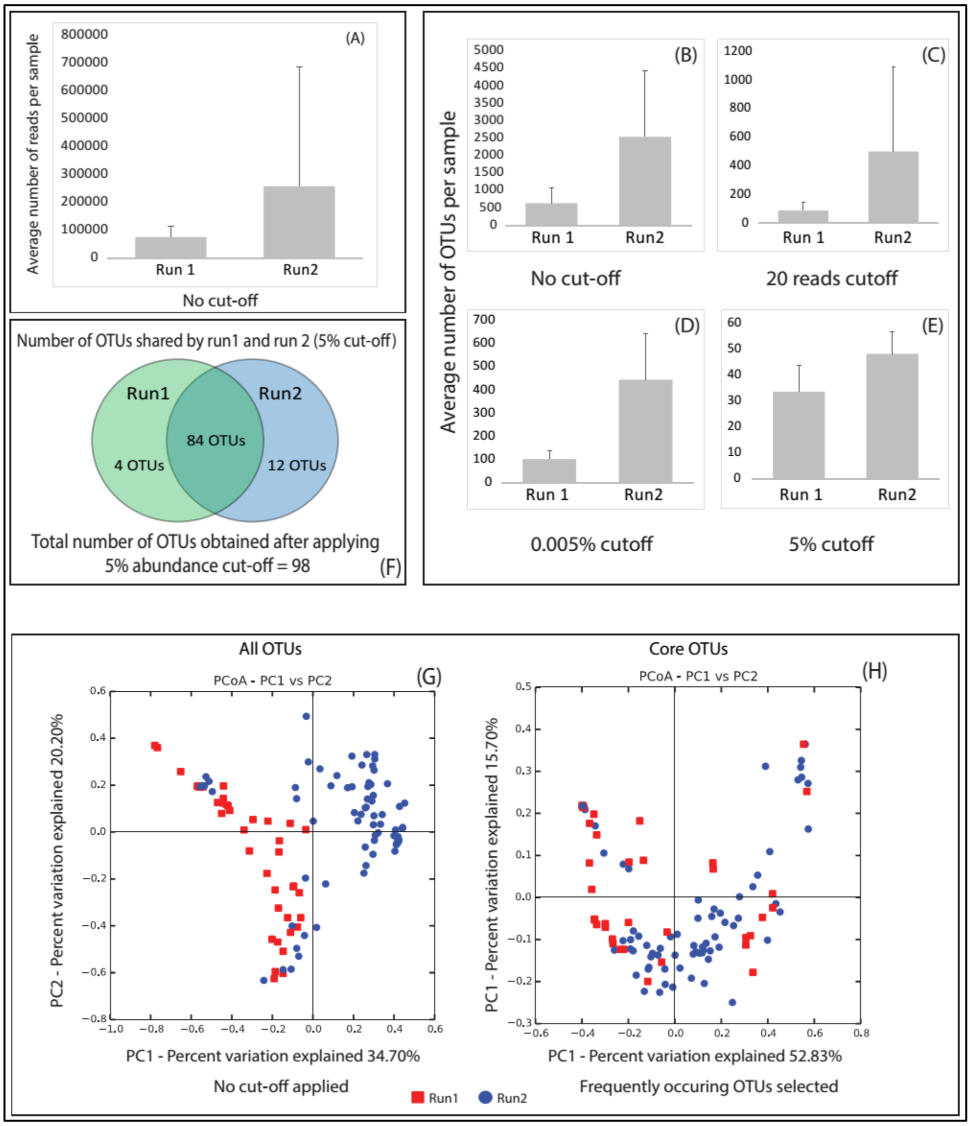

Figure S3: Total relative abundance of the 5 most abundant bacterial OTUs in each sample. Each point represents data for an individual sample (butterfly or dietary resource). Samples are colored by butterfly species. The dotted line shows the average relative abundance (0.86) contributed by the top 5 bacterial OTUs across all 130 samples.

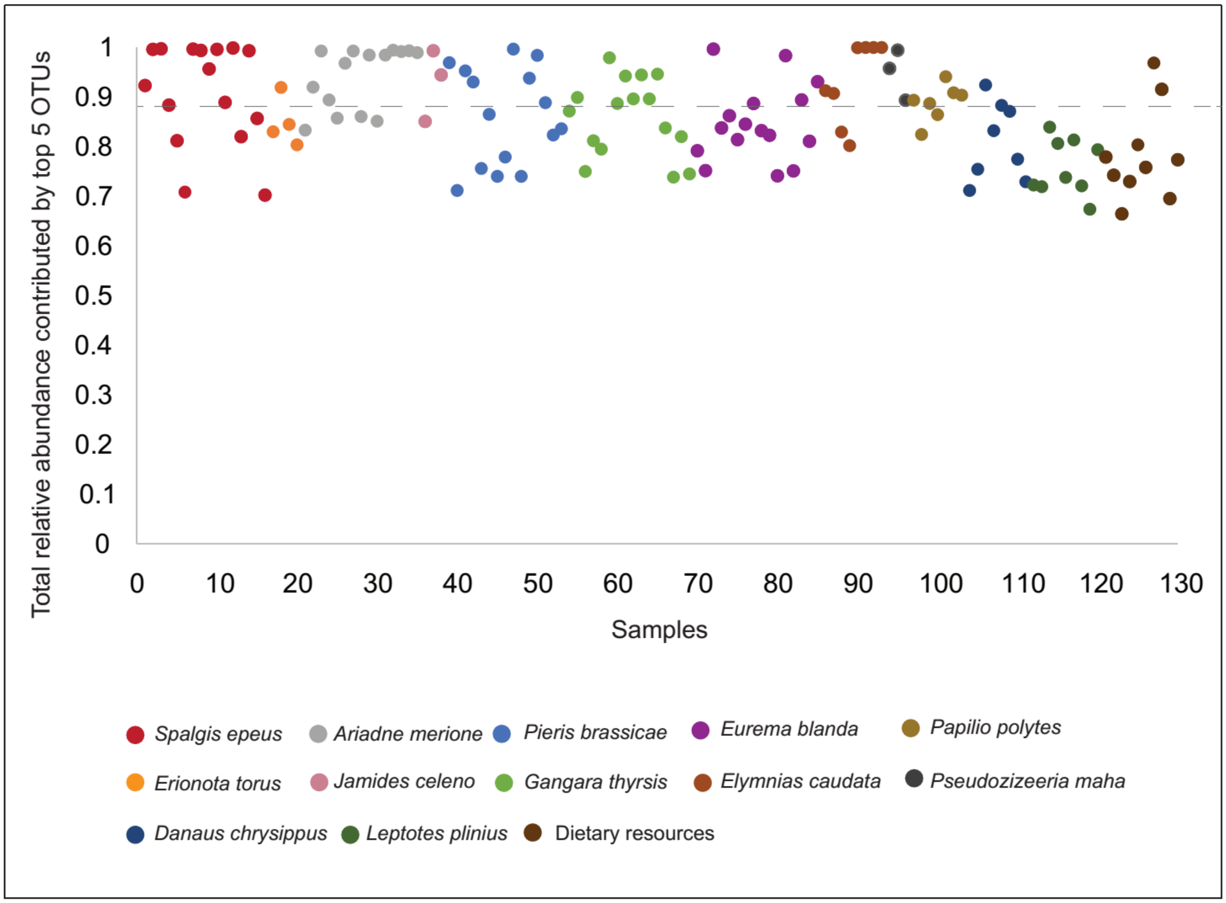

Figure S4: **Variation in total number of bacterial OTUs across samples.** Boxplots represent the number of bacterial OTUs detected in samples after applying a 5% abundance cut-off. Each panel represents a butterfly species or dietary resource.

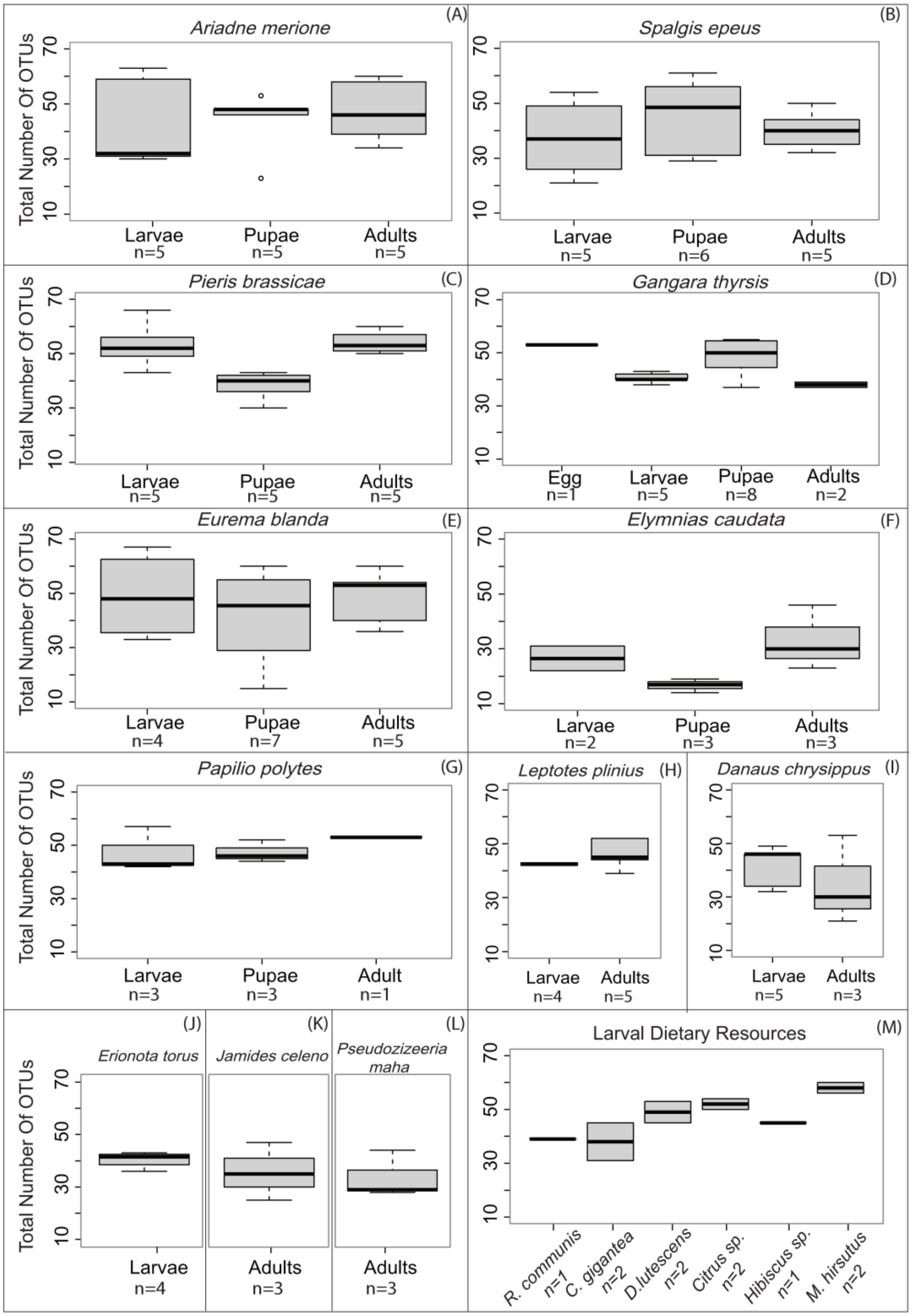

Figure S5: Diversity indices for bacterial communities. Boxplots show multiple diversity indices for pooled larvae, pupae or adult samples across host species, for bacterial communities constructed after applying 3 different rare-OTU cutoffs. Within each panel, we compared diversity indices across life stages using non-parametric Kruskal-Wallis tests. For larvae, n= 42; for pupae, n=37; for adults, n=40.

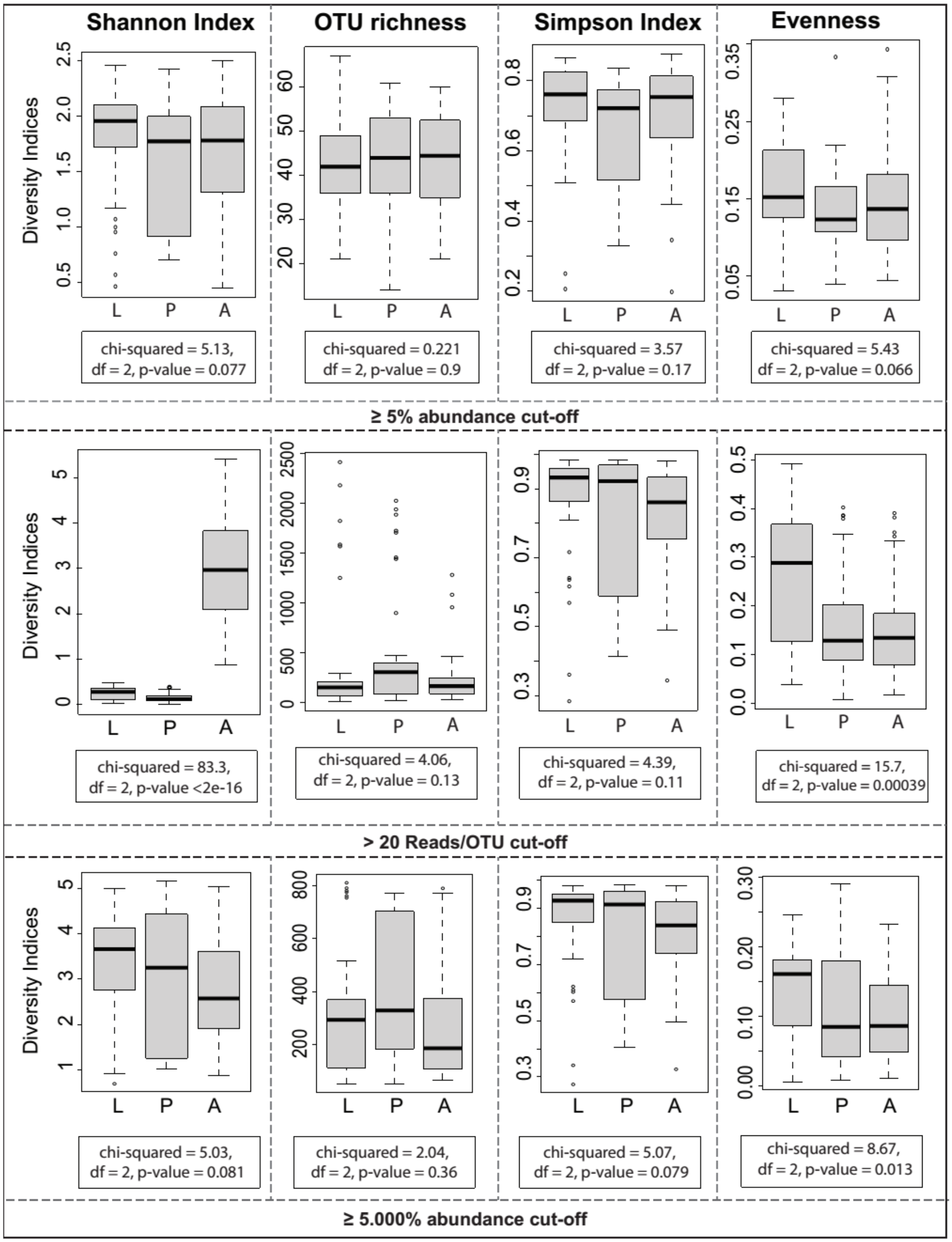

Figure S6: **Host-level individual variation in bacterial community composition across butterfly life stages**. Heat maps represent the average relative abundance (scaled from 0 to 1) of the 5 most abundant bacterial OTUs across developmental stages of each butterfly species. Each panel shows data for replicate host individuals for each developmental stage of a butterfly species. Samples (replicates) that were processed in sequencing Run1 are highlighted in grey.

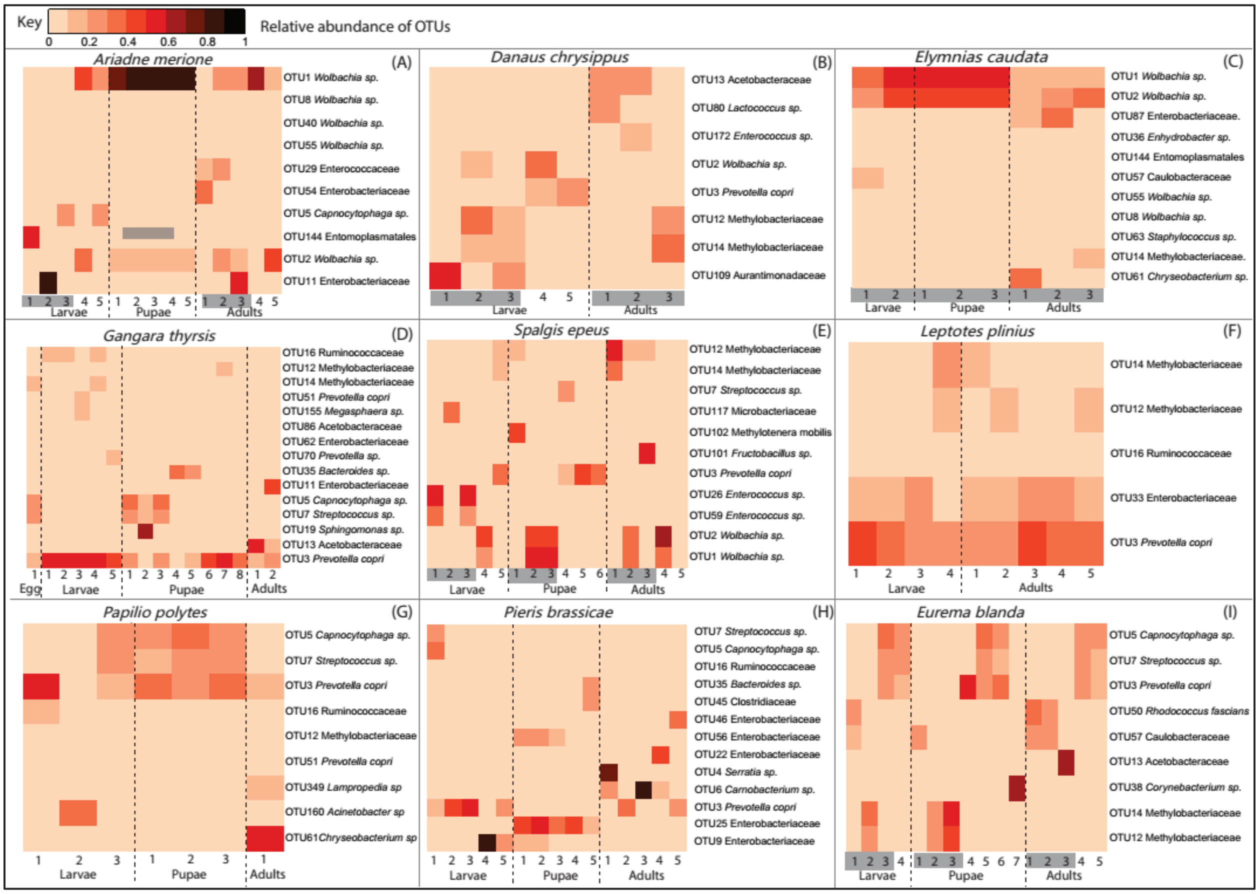

Figure S7: Individual level variation in host bacterial communities. Bar plots show the coefficient of variation (standard deviation / mean) calculated for relative abundance of the top 5 bacterial OTUs across replicate hosts within a developmental stage, for each host species. **(A)** Data for host species that show significant variation in bacterial communities across development (PERMANOVA, $p<0.05$) **(B)** Data for host species that do not show significant developmental variation in bacterial communities (PERMANOVA, $p>0.05$). CV values were not significantly different across the two groups of species shown in panels A and B (separate Student's t-tests comparing the two groups for larvae, pupae, adults or all samples; $p>0.05$ in each case).

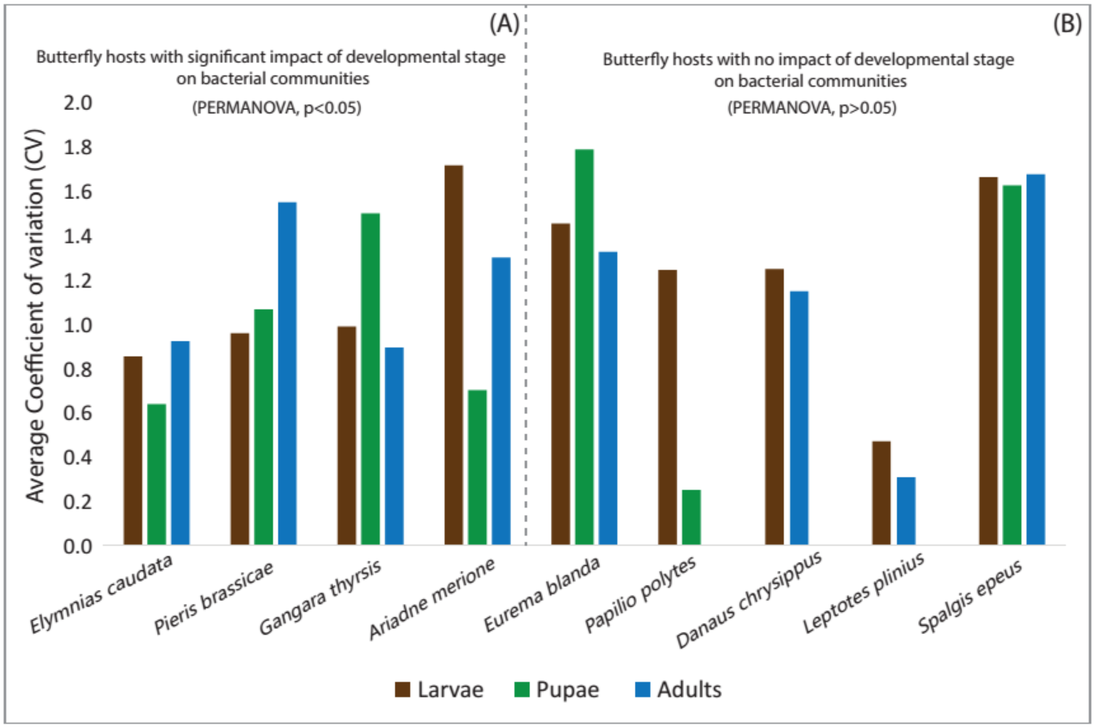

Figure S8: Variation in bacterial communities across butterfly host species. Panels show Constrained Analysis of Principal Coordinates (CAP) of butterfly host species (all developmental stages pooled) on the composition and relative abundance of bacterial OTUs after applying a 5% abundance cut-off. In each panel, different colors and symbols indicate distinct butterfly species. Axis labels indicate the proportion of between-group variance (%) explained by the first two linear discriminants (LD1 and LD2). Ellipses represent 95% confidence intervals. We observe a significant effect of host species ($p<0.05$, multivariate ANOVA).

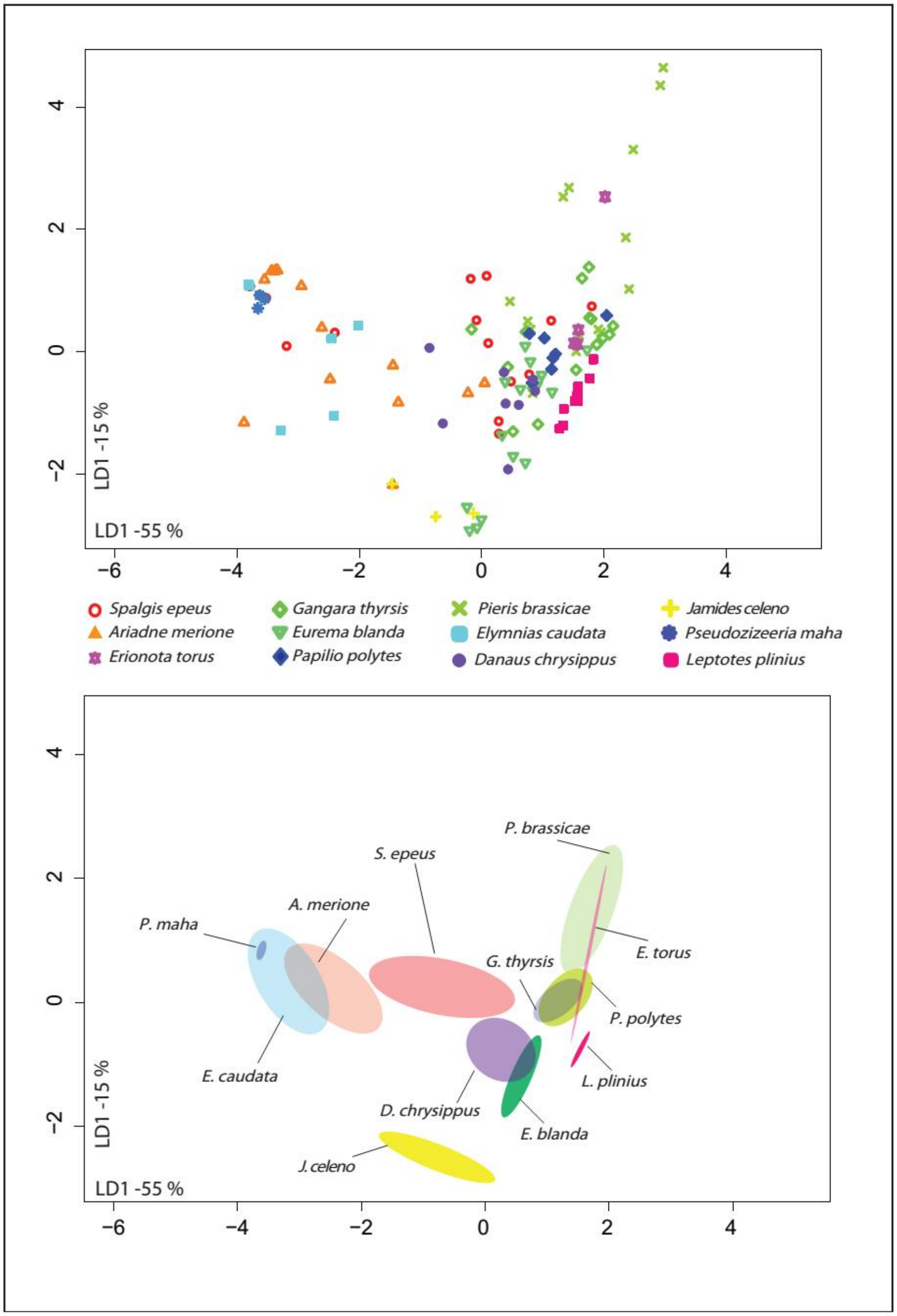

Figure S9: Variation in bacterial communities of larvae across butterfly host families, except Papilionidae. Panels show Constrained Analysis of Principal Coordinates (CAP) of larvae based on the composition and relative abundance of bacterial OTUs after applying a 5% abundance cut-off. We pooled all individuals belonging to a butterfly taxonomic family, regardless of their species. In each panel, different colors and symbols indicate distinct butterfly families. Axis labels indicate the proportion of between-group variance (%) explained by the first two linear discriminants (LD1 and LD2). Ellipses represent 95% confidence intervals. For each panel, we observe a significant effect of host family (p<0.05, multivariate ANOVA).

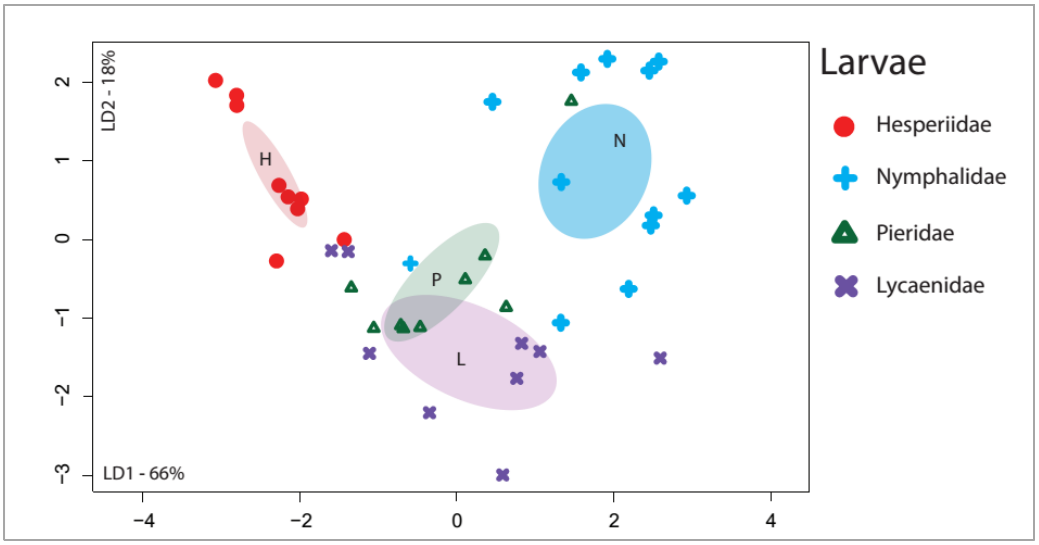

Figure S10: Variation in bacterial communities of developmental stages across butterfly host species, excluding *Wolbachia*. Panels show Constrained Analysis of Principal Coordinates (CAP) of larvae (A), pupae (B) and adults (C) based on the composition and relative abundance of bacterial OTUs after applying a 5% abundance cut-off. In each panel, different colors and symbols indicate distinct butterfly species. Axis labels indicate the proportion of between-group variance (%) explained by the first two linear discriminants (LD1 and LD2). Ellipses represent 95% confidence intervals. For each panel, we observe a significant effect of host species (p<0.05, multivariate ANOVA).

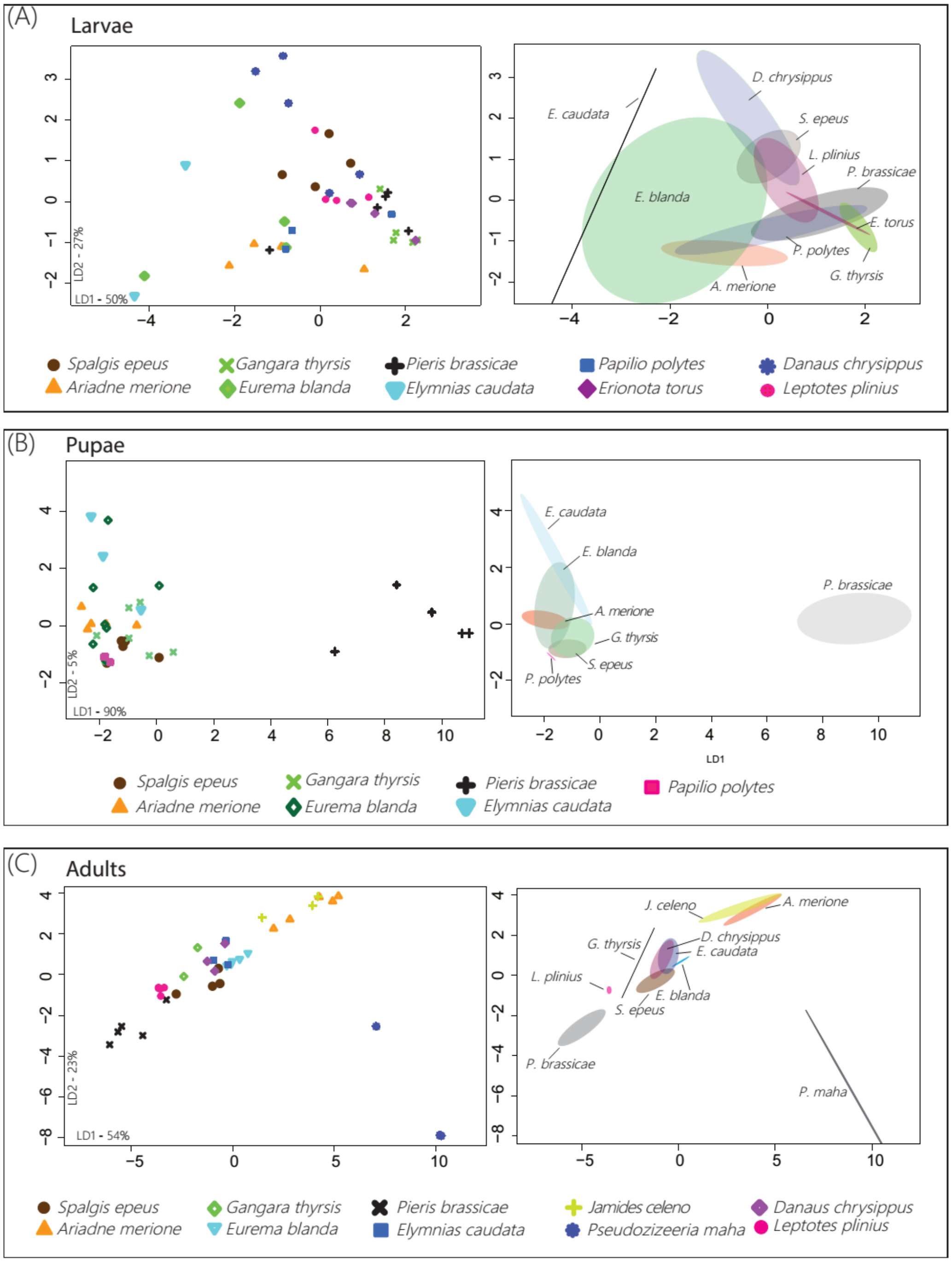

Figure S11: Variation in bacterial communities across butterfly host families, excluding *Wolbachia*. Panels show Constrained Analysis of Principal Coordinates (CAP) of larvae, pupae and adults based on the composition and relative abundance of bacterial OTUs after applying a 5% abundance cut-off. We pooled all individuals belonging to a butterfly taxonomic family, regardless of their species. In each panel, different colors and symbols indicate distinct butterfly families. Axis labels indicate the proportion of between-group variance (%) explained by the first two linear discriminants (LD1 and LD2). Ellipses represent 95% confidence intervals. For each panel, we observe a significant effect of host family ($p<0.05$, multivariate ANOVA), except larvae ($p = 0.06$).

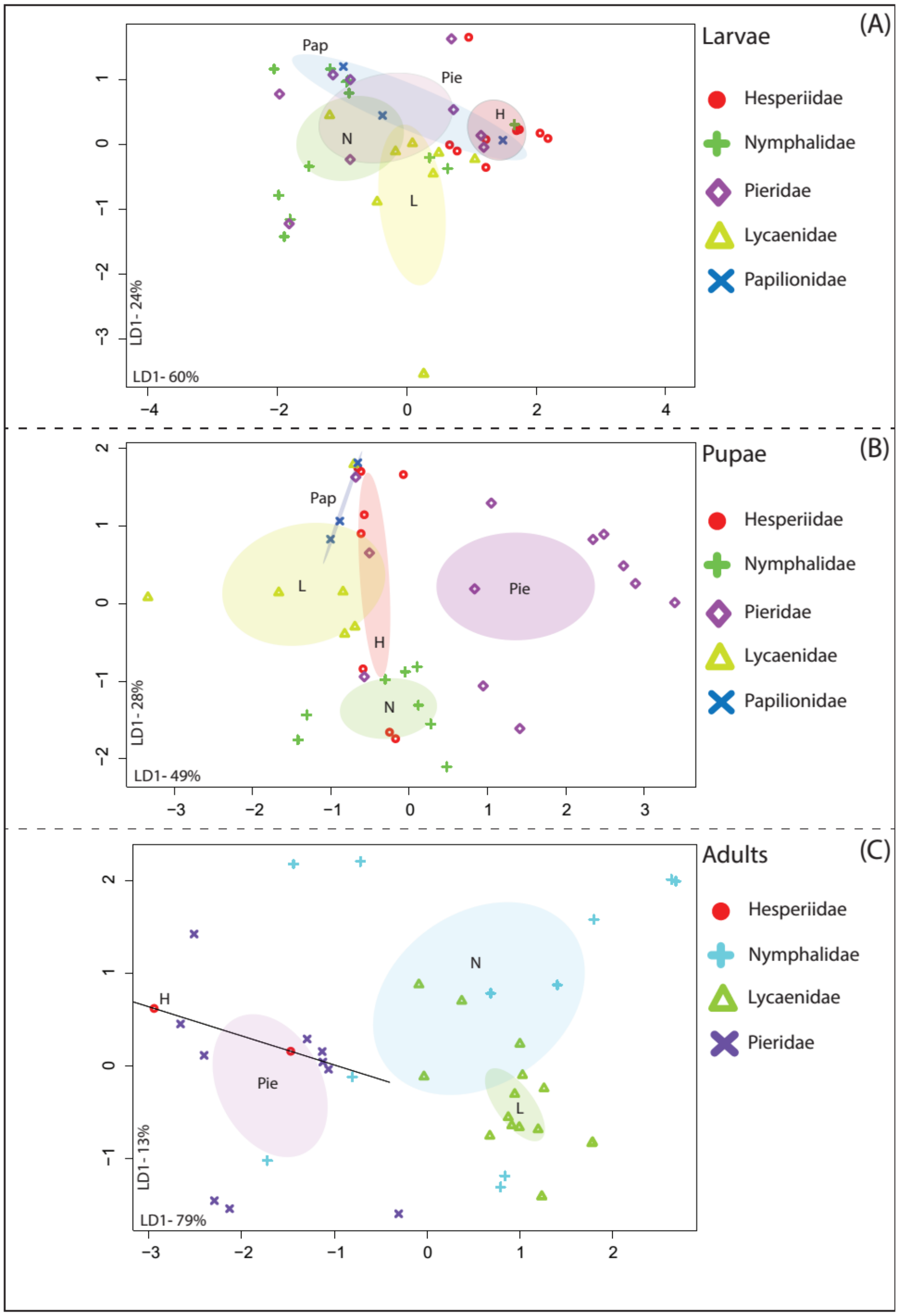

Figure S12: Variation in larval bacterial communities across host species, after excluding *E. torus* larvae. The plot shows Constrained Analysis of Principal Coordinates (CAP) of larvae based on the composition and relative abundance of bacterial OTUs after applying a 5% relative abundance cut-off. Colors and symbols indicate distinct butterfly species. Ellipses represent 95% confidence intervals. Axes labels indicate the proportion of between-group variance (%) explained by the first two linear discriminants (LD1 and LD2). We observed a significant effect of host species (p<0.05, MANOVA).

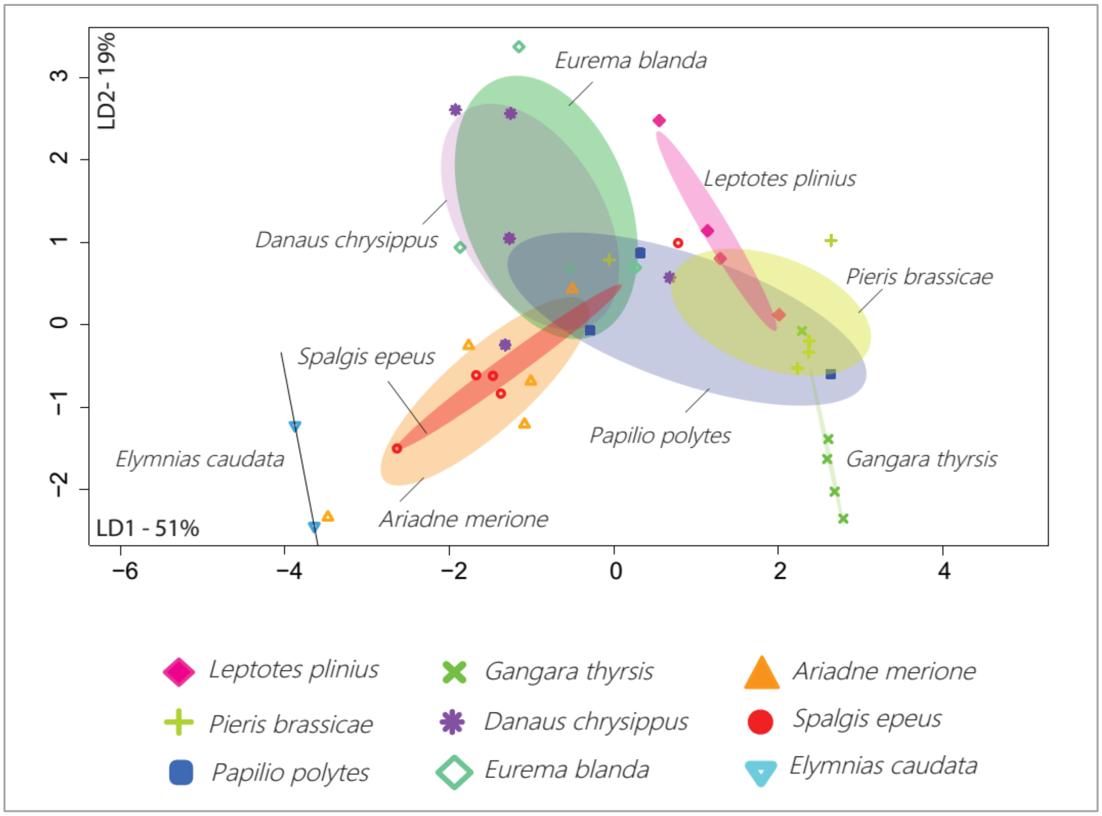

Figure S13: Variation in larval bacterial communities across host species. Plots show unconstrained analysis of Principal Coordinates (PCoA) of larvae, pupae and adults based on the phylogenetic distance between bacterial OTUs, after applying a 5% relative abundance cut-off **(S12A)**, core OTU cut-off **(S12B)** or no cut-off **(S12C)**. Colors and symbols indicate butterfly species. Axes labels indicate the proportion of variance (%) explained by the first two principle coordinates. We observed a significant effect of host species (p<0.05, PERMANOVA, Table S5).

Figure S13A:

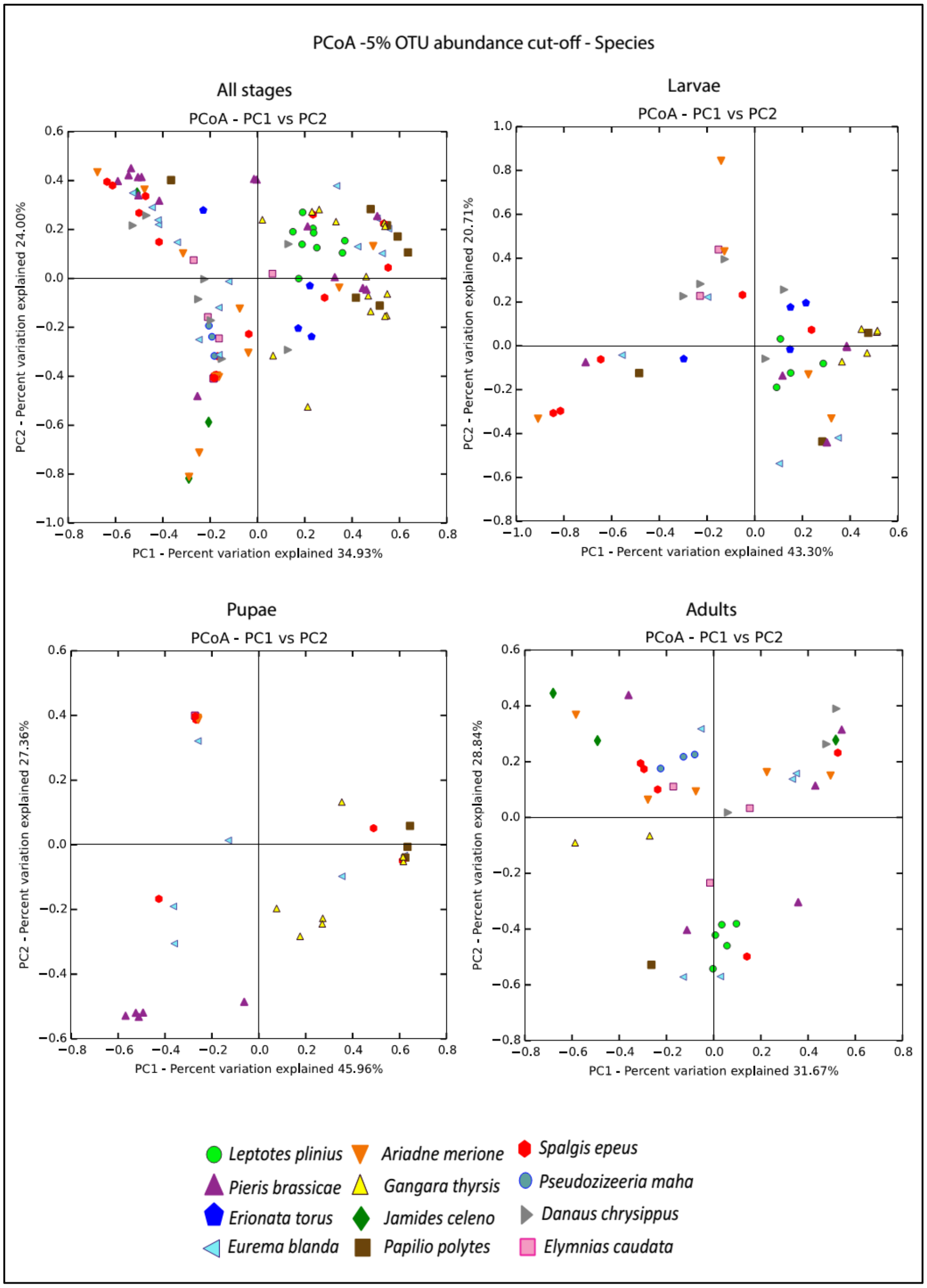

Figure S13B:

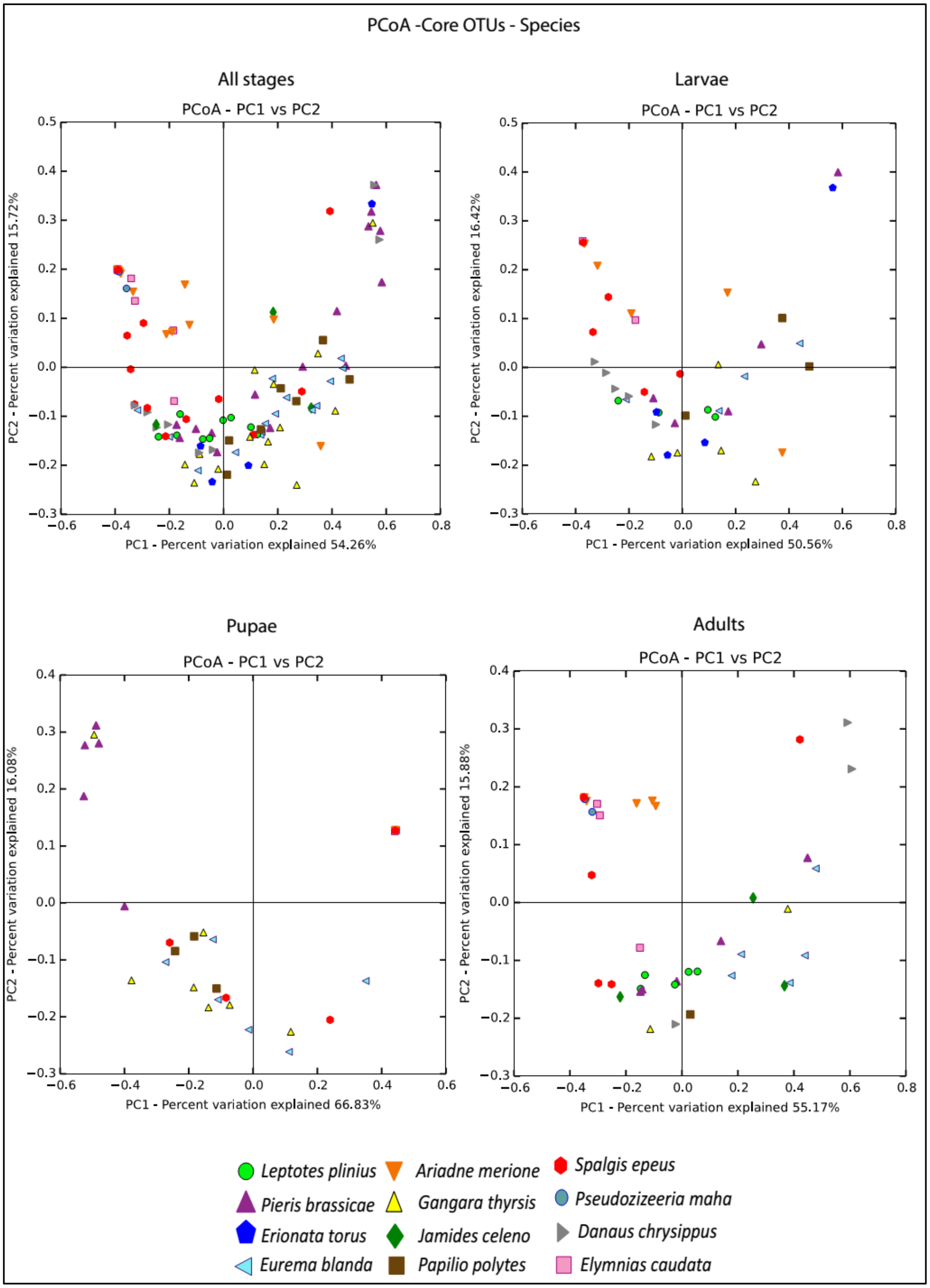
PCoA -Core OTUs - Species
All stages
PCoA - PC1 vs PC2
PC1 - Percent variation explained 54.26%
PC2 - Percent variation explained 15.72%
Larvae
PCoA - PC1 vs PC2
PC1 - Percent variation explained 50.56%
PC2 - Percent variation explained 16.42%
Pupae
PCoA - PC1 vs PC2
PC1 - Percent variation explained 66.83%
PC2 - Percent variation explained 16.08%
Adults
PCoA - PC1 vs PC2
PC1 - Percent variation explained 55.17%
PC2 - Percent variation explained 15.88%
Leptotes plinius
Ariadne merione
Spalgis epeus
Pieris brassicae
Gangara thyrsis
Pseudozizeeria maha
Erionata torus
Jamides celeno
Danaus chrysippus
Eurema blanda
Papilio polytes
Elymnias caudata

Figure S13C:

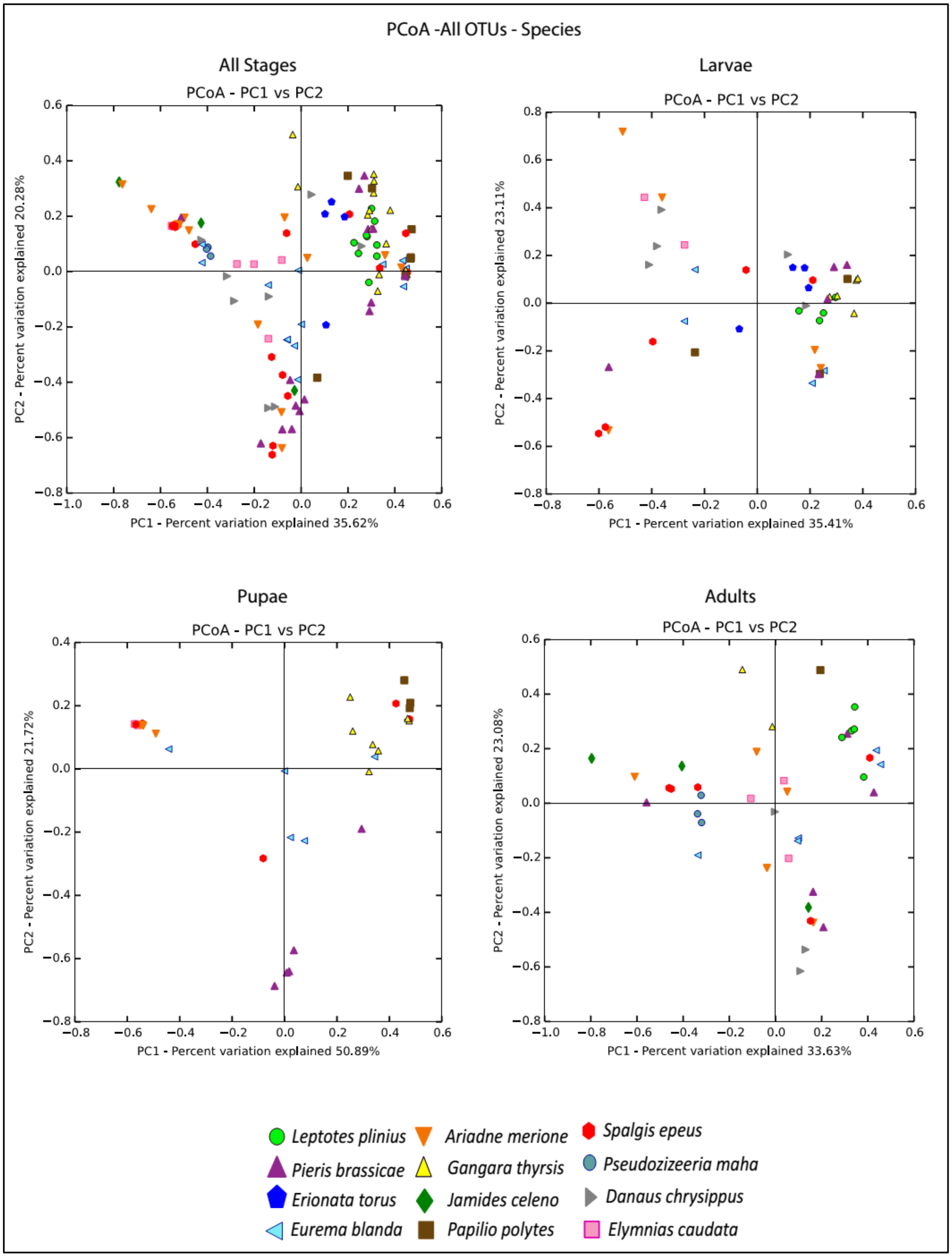
PCoA -All OTUs - Species
All Stages
PCoA - PC1 vs PC2
PC1 - Percent variation explained 35.62%
PC2 - Percent variation explained 20.28%
Larvae
PCoA - PC1 vs PC2
PC1 - Percent variation explained 35.41%
PC2 - Percent variation explained 23.11%
Pupae
PCoA - PC1 vs PC2
PC1 - Percent variation explained 50.89%
PC2 - Percent variation explained 21.72%
Adults
PCoA - PC1 vs PC2
PC1 - Percent variation explained 33.63%
PC2 - Percent variation explained 23.08%
Leptotes plinius
Ariadne merione
Spalgis epeus
Pieris brassicae
Gangara thyrsis
Pseudozizeeria maha
Erionata torus
Jamides celeno
Danaus chrysippus
Eurema blanda
Papilio polytes
Elymnias caudata

Figure S14: Variation in larval bacterial communities across host families. Plots shows unconstrained analysis of Principal Coordinates (PCoA) of larvae, pupae and adults based on the phylogenetic distance between bacterial OTUs after applying a 5% relative abundance cut-off **(S13A)**, core OTU cut-off **(S13B)** and no cut-off **(S13C)**. Colors and symbols indicate butterfly families. Axes labels indicate the proportion of variance (%) explained by the first principle coordinates. We observed a significant effect of host species ($p<0.05$, PERMANOVA, Table S5).

Figure S14A:

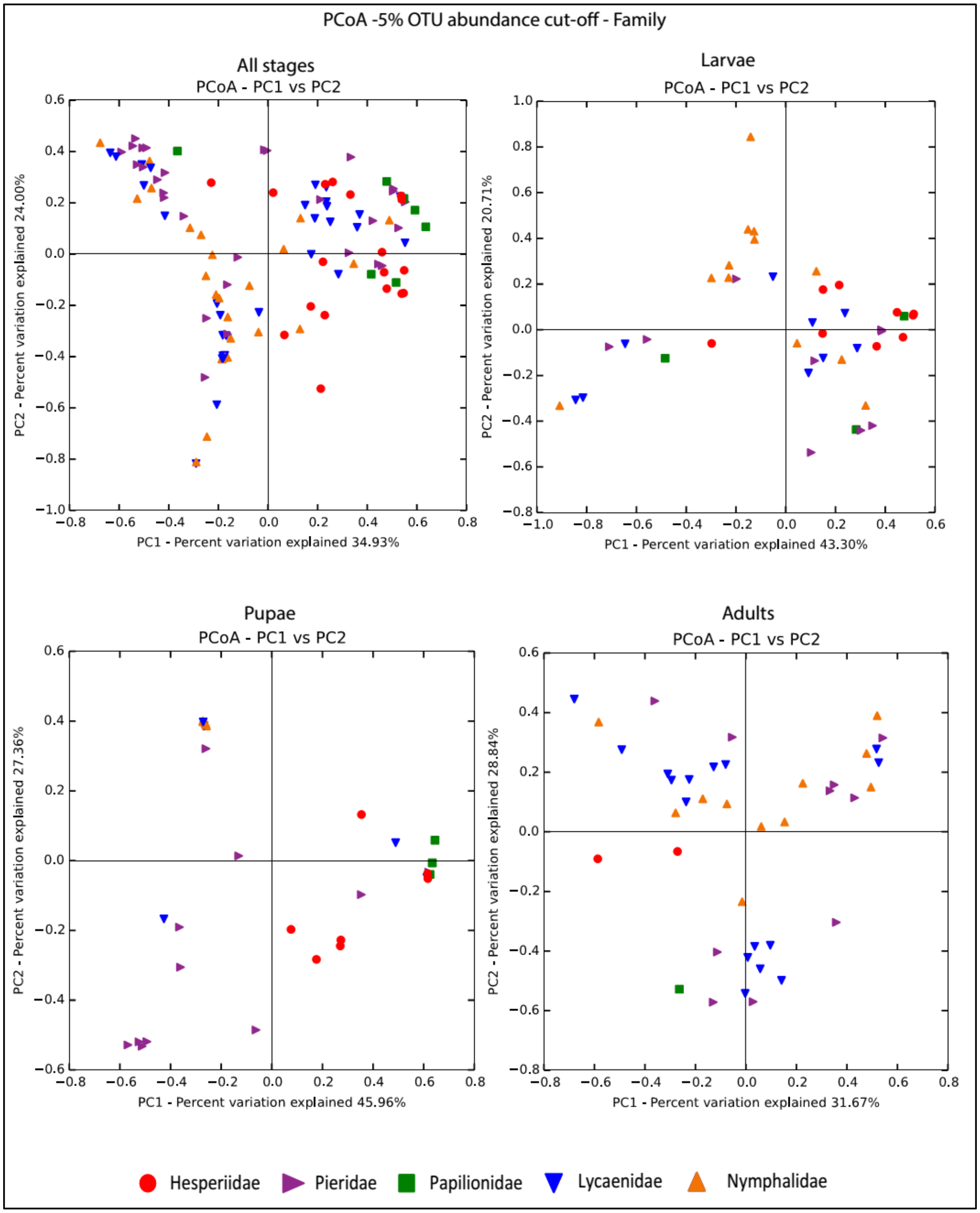

142 Figure S14B:

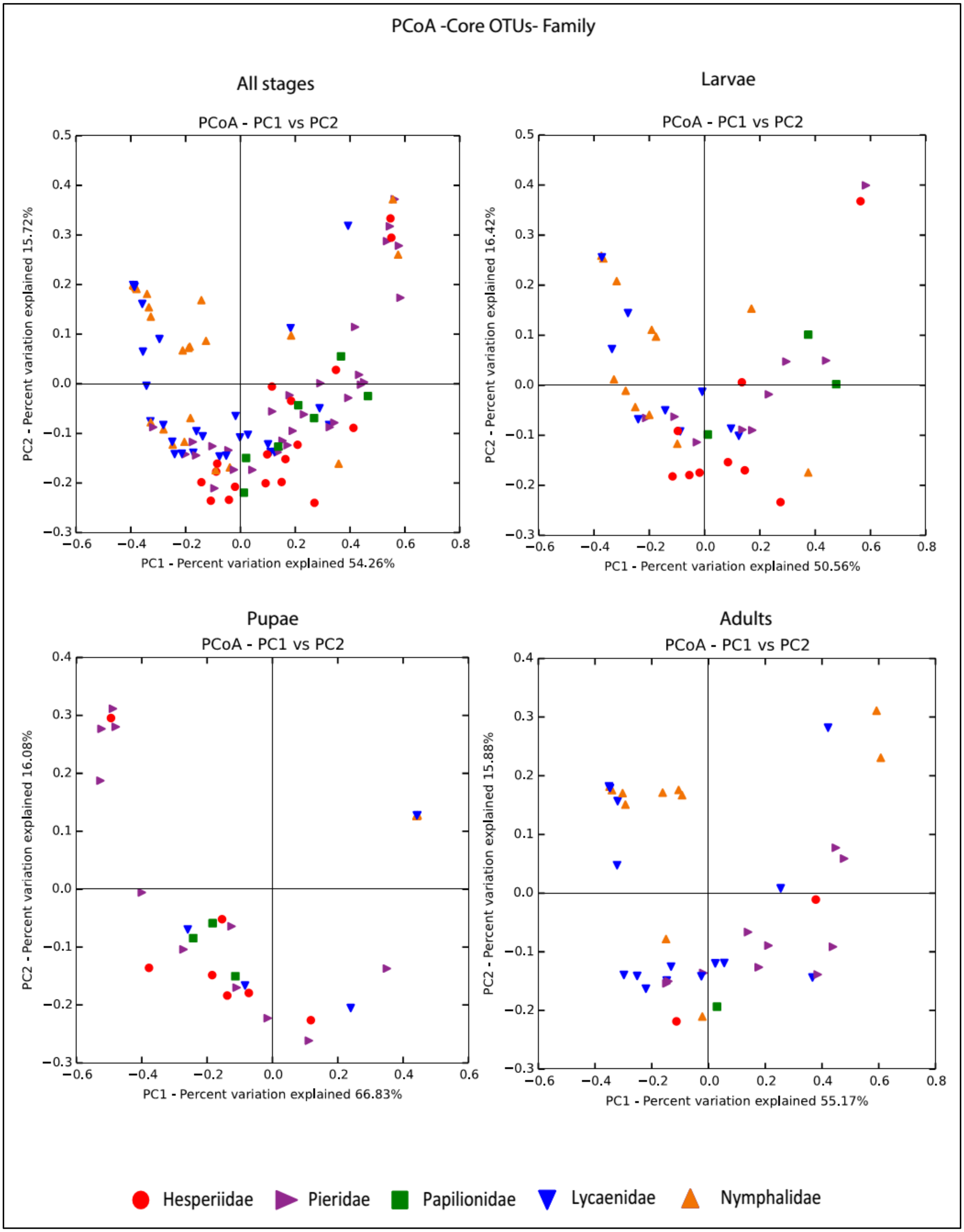

143

Figure S14C:

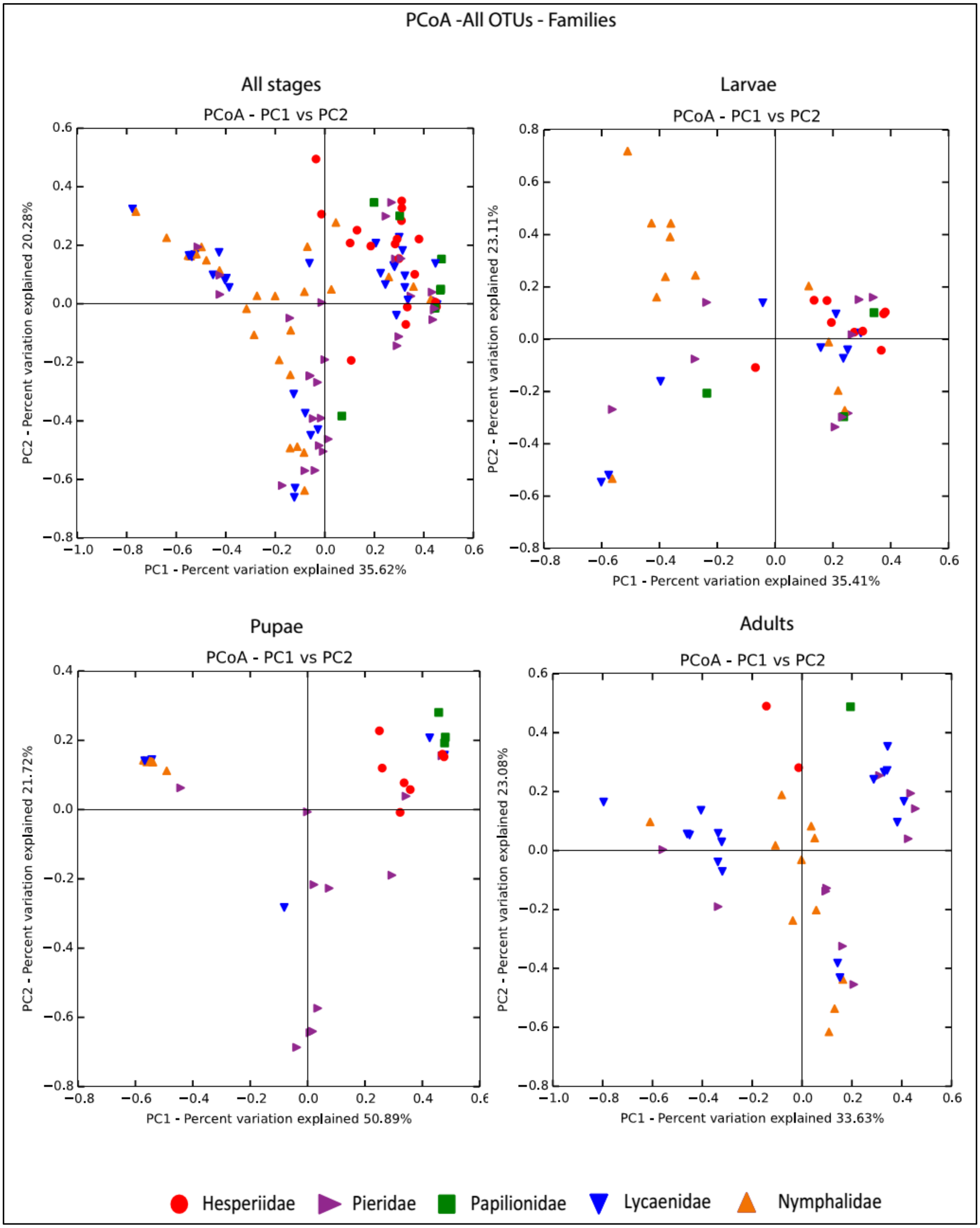
PCoA -All OTUs - Families
All stages
PCoA - PC1 vs PC2
PC1 - Percent variation explained 35.62%
PC2 - Percent variation explained 20.28%
Larvae
PCoA - PC1 vs PC2
PC1 - Percent variation explained 35.41%
PC2 - Percent variation explained 23.11%
Pupae
PCoA - PC1 vs PC2
PC1 - Percent variation explained 50.89%
PC2 - Percent variation explained 21.72%
Adults
PCoA - PC1 vs PC2
PC1 - Percent variation explained 33.63%
PC2 - Percent variation explained 23.08%
Hesperiidae
Pieridae
Papilionidae
Lycaenidae
Nymphalidae
