# Supplementary material for: Dietary and developmental shifts in butterfly-associated bacterial communities
Source: R Soc Open Sci. 2018 May 30;5(5):171559. doi: 10.1098/rsos.171559 (PMC5990769; doi:10.1098/rsos.171559)
Supplement: Supplementary Tables [file rsos171559supp3.pdf]

DIETARY AND DEVELOPMENTAL SHIFTS IN BUTTERFLY-ASSOCIATED BACTERIAL COMMUNITIES

Kruttika Phalnikar*, Krushnamegh Kunte and Deepa Agashe*

National Centre for Biological Sciences (NCBS), GKVK Campus, Bellary Road, Bangalore, India 560065

*Corresponding authors:

kruttikap@ncbs.res.in

dagashe@ncbs.res.in

SUPPLEMENTARY TABLES

Table S1: Variation in bacterial communities across different developmental stages of butterfly species. Tables S1A, S1B and S1C show the output of PERMANOVA analysis (with 10,000 permutations), carried out to test the variation in bacterial community structure across butterfly development. **Table S1A** shows the variation in bacterial communities across larvae and adults, with different OTU filtering cut-offs. **Table S1B** shows the variation in bacterial communities across larvae, pupae and adults, with different OTU filtering cut-offs. **Table S1C** shows the impact of sequencing runs on variation in bacterial communities across host development. For 3 out of 12 host butterfly species, different developmental stages were split across these two sequencing runs. In Tables S1A, S1B and S1C, R^2 values represent the proportion of variation explained by each treatment, from a PERMANOVA analysis. **Table S1D** shows the output of CAPdiscrim ordination performed for host developmental stages after applying a 5% abundance cut-off on bacterial OTUs (see methods). Percent classification success (CS) indicates the proportion of successful assignment of individuals to their original clusters, based on the discriminant functions. Larger values of CS indicate better cluster separation, while lower values suggest admixed groups. LD1 and LD2 are the linear discriminants that best describe the classification performed by linear discriminant analysis and represent between-group variation explained.

Table S1A: Variation in bacterial communities across Larvae and Adults

Host species	Top 5 OTUs		≥5% relative abundance		> 20 Reads per OTU		≥0.005% relative abundance		Core OTUs	
	p value	R^2 value	p value	R^2 value	p value	R^2 value	p value	R^2 value	p value	R^2 value
Spalgis epeus	0.43	0.11	0.35	0.12	0.14	0.16	0.20	0.14	0.98	0.01
Leptotes plinius	0.96	0.01	0.88	0.06	0.11	0.59	0.62	0.11	0.82	0.04
Pieris brassicae	0.06	0.21	0.05	0.22	0.18	0.05	0.06	0.19	0.03	0.25
Eurema blanda	0.91	0.05	0.90	0.05	0.87	0.05	0.89	0.04	0.49	0.09
Ariadne merione	0.31	0.13	0.22	0.15	0.13	0.28	0.27	0.13	0.10	0.20
Elymnias caudata	0.20	0.37	0.20	0.32	0.34	0.20	0.30	0.31	0.80	0.10
Danaus chrysippus	0.07	0.30	0.07	0.28	0.04	0.26	0.05	0.27	0.05	0.46
Gangara thyrsis	0.04	0.61	0.04	0.53	0.04	0.31	0.04	0.32	0.23	0.22

Table S1B: Variation in bacterial communities across Larvae, Pupae and Adults

Host Species	Top 5 abundant OTUs		≥5% relative abundance		> 20 Reads per OTU		≥0.005% relative abundance		Core OTUs	
	p value	R^2 value	p value	R^2 value	p value	R^2 value	p value	R^2 value	p value	R^2 value
Spalgis epeus	0.4125	0.1377	0.3672	0.1418	0.2711	0.1530	0.2970	0.1508	0.9803	0.02913
Pieris brassicae	0.0002	0.4653	0.0002	0.4311	0.0002	0.3586	0.0001	0.3738	0.0026	0.47083
Eurema blanda	0.8965	0.0703	0.8893	0.0755	0.9219	0.0735	0.9119	0.0704s	0.5093	0.11845
Ariadne merione	0.0014	0.3257	0.0011	0.3403	0.0009	0.3526	0.0005	0.3569	0.009599	0.30789
Elymnias caudata	0.0407	0.5477	0.0418	0.4852	0.0188	0.4480	0.0304	0.4904	0.24477	0.4736
Gangara thyrsis	0.0026	0.3477	0.0057	0.3260	0.0062	0.2739	0.0164	0.2576	0.06119	0.23332
Papilio polytes	0.3000	0.2540	0.3000	0.2696	0.3000	0.2955	0.3000	0.3037	0.6	0.17947

Table S1C: Effect of sequencing run on variation in bacterial communities across host development				
Host species	Comparison across larvae, pupae and adults		Comparison across larvae, pupae and adults	
	Run 1		Run 2	
	p Value	R^2 Value	p Value	R^2 Value
Spalgis epeus	0.05	0.41	0.18	0.44
Ariadne merione	No pupae sequenced in run 1		0.03	0.56
Eurema blanda	0.59	0.24	0.52	0.26
Host species	Comparison across larvae and adults		Comparison across larvae and adults	
	Run 1		Run 2	
	p Value	R^2 Value	p Value	R^2 Value
Spalgis epeus	0.1	0.39	0.66	0.26
Ariadne merione	0.3	0.38	0.50	0.21
Eurema blanda	0.80	0.17	0.59	0.27

Table S1D: Variation in bacterial communities across Larvae, Pupae, and Adults (CAPdiscrim)				
Species	Classification success	LD1	LD2	MANOVA (p)
Spalgis epeus	50%	65%	34%	0.34
Leptotes plinius	44%	100%	0%	0.85
Pieris brassicae	86%	96%	4%	<0.0001
Eurema blanda	50%	98%	1%	0.78
Ariadne merione	80%	63%	36%	0.0006
Elymnias caudata	63%	100%	0%	0.0265
Danaus chrysippus	87%	100%	0%	0.07
Gangara thyrsis	86%	0.88	11%	<0.0001
Papilio polytes	83%	100%	0%	0.36

Tables S2: Individual variation in bacterial OTU abundance. The tables (A-I) show the mean relative abundance of the top 5 bacterial OTUs and standard deviation (SD) in relative abundance across replicates of larvae, pupae and adults of each host species.

(A) *D. chrysippus*	Larvae		Adults	
	**Mean**	**SD**	**Mean**	SD
OTU109 Aurantimonadaceae	0.1607	0.2227	0.0000	0.0000
OTU2 *Wolbachia sp.*	0.0955	0.1400	0.0000	0.0000
OTU3 *Prevotella copri*	0.0928	0.1350	0.0000	0.0000
OTU12 *Methylobacteriaceae*	0.1429	0.1406	0.0733	0.1264
OTU14 *Methylobacteriaceae*	0.0966	0.0628	0.1029	0.1781
OTU13 Acetobacteraceae	0.0002	0.0002	0.1773	0.1539
OTU80 *Lactococcus sp.*	0.0001	0.0001	0.1054	0.1456
OTU172 *Enterococcus sp.*	0.0001	0.0001	0.0761	0.0883

(B) *E. caudata*	Larvae		Pupae		Adults	
	Mean	SD	Mean	SD	Mean	SD
OTU55 *Wolbachia sp.*	0.0076	0.0036	0.0093	0.0012	0.0025	0.0016
OTU63 *Staphylococcus sp.*	0.0003	0.0004	0.0004	0.0008	0.0096	0.0087
OTU8 *Wolbachia sp.*	0.0002	0.0003	0.0004	0.0000	0.0001	0.0000
OTU57 *Caulobacteraceae*	0.0506	0.0713	0.0001	0.0002	0.0009	0.0015
OTU36 *Enhydrobacter sp.*	0.0264	0.0369	0.0000	0.0000	0.0078	0.0134
OTU144 *Entomoplasmatales*	0.0256	0.0362	0.0000	0.0000	0.0000	0.0000
OTU1 *Wolbachia sp.*	0.4216	0.1552	0.5134	0.0161	0.1863	0.0167
OTU2 *Wolbachia sp.*	0.3707	0.1211	0.4759	0.0182	0.2134	0.0889
OTU14 Methylobacteriaceae	0.0031	0.0042	0.0000	0.0000	0.0425	0.0736
OTU87 Enterobacteriaceae	0.0000	0.0000	0.0000	0.0000	0.1625	0.1900
OTU61 *Chryseobacterium*	0.0000	0.0000	0.0000	0.0000	0.1330	0.2303

(C) *A. merione*	Larvae		Pupae		Adults	
	Mean	SD	Mean	SD	Mean	SD
OTU54 Enterobacteriaceae	0.0010	0.0014	0.0001	0.0000	0.0762	0.1686
OTU29 Enterococcaceae	0.0001	0.0001	0.0000	0.0001	0.0899	0.0868
OTU11 Enterobacteriaceae	0.1774	0.3962	0.0001	0.0002	0.1129	0.2523
OTU1 *Wolbachia sp.*	0.1632	0.2020	0.7970	0.0257	0.2796	0.2189
OTU2 *Wolbachia sp.*	0.0869	0.1673	0.1157	0.0153	0.1991	0.1422
OTU5 *Capnocytophaga sp.*	0.0984	0.1374	0.0000	0.0001	0.0204	0.0280
OTU144 *Entomoplasmatales*	0.1119	0.2501	0.0000	0.0000	0.0000	0.0000
OTU8 *Wolbachia sp.*	0.0073	0.0094	0.0611	0.0038	0.0104	0.0228
OTU40 *Wolbachia sp.*	0.0206	0.0437	0.0108	0.0021	0.0015	0.0034
OTU55 *Wolbachia sp.*	0.0034	0.0060	0.0059	0.0010	0.0041	0.0014

(D) *G. thyrsis*	Larvae		Pupae		Adults	
	Mean	SD	Mean	SD	SD	Mean
OTU62 Enterobacteriaceae	0.0012	0.0013	0.0014	0.0017	0.0335	0.0466
OTU13 Acetobacteraceae	0.0136	0.0210	0.0011	0.0023	0.3678	0.3116
OTU86 Acetobacteraceae	0.0022	0.0028	0.0008	0.0015	0.0405	0.0237
OTU11 Enterobacteriaceae	0.0157	0.0236	0.0000	0.0001	0.2248	0.3165
OTU3 *Prevotella copri*	0.5279	0.0287	0.2462	0.1711	0.1944	0.0860
OTU16 Ruminococcaceae	0.0954	0.0644	0.0573	0.0163	0.0196	0.0040
OTU70 Paraprevotellaceae, *Prevotella*	0.0617	0.0803	0.0191	0.0172	0.0087	0.0042
OTU51 Prevotella copri	0.0589	0.0576	0.0128	0.0071	0.0147	0.0127
OTU155 *Megasphaera sp.*	0.0361	0.0769	0.0007	0.0011	0.0000	0.0000
OTU5 *Capnocytophaga sp.*	0.0011	0.0009	0.0985	0.1491	0.0061	0.0084
OTU7 *Streptococcus sp.*	0.0112	0.0103	0.0863	0.1191	0.0059	0.0078
OTU35 *Bacteroides sp.*	0.0021	0.0010	0.0829	0.1532	0.0037	0.0048
OTU19 *Sphingomonas sp.*	0.0000	0.0000	0.0776	0.2128	0.0000	0.0000

(E) *L. plinius*	Larvae		Adults	
	Mean	SD	Mean	SD
OTU3 *Prevotella copri*	0.3248	0.1000	0.3362	0.0898
OTU33 Enterobacteriaceae	0.1614	0.0492	0.1808	0.0509
OTU14 Methylobacteriaceae	0.1074	0.0736	0.0839	0.0187
OTU12 Methylobacteriaceae	0.0922	0.0656	0.0827	0.0315
OTU16 Ruminococcaceae	0.0563	0.0198	0.0559	0.0222

(F) *S. epeus*	Larvae		Pupae		Adults	
	Mean	SD	Mean	SD	SD	Mean
OTU14 Methylobacteriaceae	0.0342	0.0567	0.0201	0.0339	0.1073	0.1588
OTU12 Methylobacteriaceae	0.0281	0.0456	0.0313	0.0478	0.1717	0.2427
OTU101 *Fructobacillus sp.*	0.0000	0.0001	0.0000	0.0000	0.1053	0.2355
OTU26 *Enterococcus sp.*	0.2345	0.3196	0.0004	0.0008	0.0000	0.0000
OTU59 *Enterococcus sp.*	0.1183	0.1615	0.0001	0.0002	0.0000	0.0000
OTU117 Microbacteriaceae	0.0618	0.1374	0.0004	0.0007	0.0001	0.0002
OTU7 *Streptococcus sp.*	0.0009	0.0013	0.0610	0.1087	0.0003	0.0007
OTU102 Methylotenera mobilis	0.0000	0.0000	0.0840	0.2032	0.0000	0.0000
OTU1 *Wolbachia sp.*	0.0616	0.0940	0.1738	0.2583	0.1471	0.1998
OTU2 *Wolbachia sp.*	0.1027	0.1887	0.1549	0.2372	0.2099	0.2971
OTU3 *Prevotella copri*	0.0810	0.1425	0.1691	0.2032	0.0099	0.0202

(G) *P. brassicae*	Larvae		Pupae		Adults	
	**Mean**	**SD**	**Mean**	**SD**	**SD**	**Mean**
**OTU46 Enterobacteriaceae**	0.0000	0.0000	0.0004	0.0007	0.0685	0.1501
**OTU6 *Carnobacterium sp.***	0.0000	0.0000	0.0000	0.0000	0.2489	0.3196
**OTU4 *Serratia sp.***	0.0000	0.0000	0.0000	0.0000	0.1465	0.3166
**OTU22 Enterobacteriaceae**	0.0000	0.0000	0.0000	0.0000	0.0958	0.2119
**OTU3 *Prevotella copri***	0.2994	0.2106	0.0208	0.0137	0.1368	0.1685
**OTU16 Ruminococcaceae**	0.0569	0.0344	0.0083	0.0145	0.0206	0.0236
**OTU7 *Streptococcus sp.***	0.0652	0.1189	0.0003	0.0004	0.0024	0.0031
**OTU5 *Capnocytophaga sp.***	0.0634	0.1346	0.0000	0.0000	0.0003	0.0004
**OTU9 Enterobacteriaceae**	0.2281	0.3664	0.0921	0.0570	0.0039	0.0058
**OTU25 Enterobacteriaceae**	0.0047	0.0064	0.3964	0.1498	0.0015	0.0026
**OTU56 Enterobacteriaceae**	0.0033	0.0045	0.1522	0.0775	0.0015	0.0021
**OTU35 *Bacteroides sp.***	0.0039	0.0042	0.0564	0.1236	0.0006	0.0009
**OTU45 Clostridiaceae**	0.0008	0.0015	0.0428	0.0946	0.0007	0.0010

(H) *P. polytes*	Larvae		Adults	
	**Mean**	**SD**	**Mean**	**SD**
**OTU5 *Capnocytophaga sp.***	0.1002	0.1730	0.3054	0.0406
**OTU3 *Prevotella copri***	0.2683	0.2365	0.2966	0.0876
**OTU7 *Streptococcus sp.***	0.0836	0.1396	0.2349	0.0373
**OTU16 Ruminococcaceae**	0.0645	0.0568	0.0610	0.0030
**OTU160 *Acinetobacter sp.***	0.1205	0.2036	0.0004	0.0003
**OTU51 *Prevotella copri***	0.0175	0.0108	0.0175	0.0031

(I) *E. blanda*	Larvae		Pupae		Adults	
	**Mean**	**SD**	**Mean**	**SD**	**SD**	**Mean**
**OTU57 Caulobacteraceae**	0.0458	0.0870	0.0364	0.0751	0.0884	0.1212
**OTU50 *Rhodococcus fascians***	0.0634	0.1254	0.0086	0.0188	0.1147	0.1607
**OTU13 Acetobacteraceae**	0.0001	0.0001	0.0001	0.0001	0.1221	0.2719
**OTU7 *Streptococcus sp.***	0.1164	0.1354	0.0628	0.1018	0.0839	0.1176
**OTU5 *Capnocytophaga sp.***	0.1425	0.1675	0.0822	0.1412	0.1072	0.1483
**OTU3 *Prevotella copri***	0.1002	0.1127	0.1581	0.1974	0.0730	0.1017
**OTU14 Methylobacteriaceae**	0.1010	0.1736	0.1321	0.2095	0.0113	0.0084
**OTU12 Methylobacteriaceae**	0.0825	0.1224	0.1112	0.1590	0.0191	0.0154
**OTU38 *Corynebacterium sp.***	0.0014	0.0016	0.0997	0.2596	0.0005	0.0006

Table S3: Comparing bacterial communities of larvae, pupae and adults across host species and families. (A) The output of Constrained Analysis of Principal Coordinates (CAP) on butterfly species (S) and families (F) based on the composition and relative abundance of bacterial OTUs. LD1 and LD2 correspond to first two linear discriminants and values represent between group variation (%) explained by each linear discriminant. Percent classification success (CS) indicates the proportion of successful reassignment of individuals to their original clusters, based on the discriminant functions. Larger values of CS indicate better cluster separation, while lower values suggest admixed groups. Analysis for "all stages" was only carried out for 5% relative abundance cutoff. **(B)** The same analysis carried out by excluding OTUs categorized as *Wolbachia* with 5% relative abundance cutoff. All comparisons were statistically significant (MANOVA, $p<0.05$) except the category - without *Wolbachia*, Larvae-Family (MANOVA, $p<0.05$).

Table S3A: With *Wolbachia*

Stage	Group	Top 5 abundant OTUs				OTUs with ≥5% relative abundance				Minimum 20 Reads per OTU				OTUs with ≥0.005% relative abundance				Core OTUs			
		LD1	LD2	Total LD	CS	LD1	LD2	Total LD	CS	LD1	LD2	Total LD	CS	LD1	LD2	Total LD	CS	LD1	LD2	Total LD	CS
Larvae	S	61	22	83	50	75	13	88	50	51	25	76	50	64	16	80	50	80	19	99	29
Larvae	F	70	16	86	52	66	18	84	55	67	17	94	60	60	22	82	60	90	7	97	43
Pupae	S	79	19	98	62	83	15	98	62	87	11	98	62	93	6	99	62	70	22	92	57
Pupae	F	87	13	100	85	87	13	100	62	82	14	96	65	83	13	96	68	88	10	98	57
Adults	S	60	23	83	59	67	18	85	56	68	25	83	74	80	9	89	64	48	26	74	49
Adults	F	79	12	91	67	74	18	92	69	80	9	89	64	70	22	92	67	67	25	92	54
All stages	S	-	-	-	-	55	15	70	44	-	-	-	-	-	-	-	-	-	-	-	-
All stages	F	-	-	-	-	77	14	91	54	-	-	-	-	-	-	-	-	-	-	-	-

Table S3B) Without *Wolbachia*

Stage	Group	OTUs with ≥5% relative abundance			
		LD1	LD2	Total LD	CS
Larvae	Species	50	27	77	36
Larvae	Family	60	24	84	50
Pupae	Species	90	5	95	51
Pupae	Family	49	28	77	57
Adult	Species	54	23	77	69
All stages	Species	38	22	60	37
All stages	Family	64	25	89	40

Table S4: Variation in bacterial communities of butterflies across host species and families. Tables show the output of PERMANOVA analysis (10,000 permutations) when variation in bacterial community structure was tested across butterfly species and families, R^2 value indicates % variation explained. Tables show the PERMANOVA output for pooled samples **(S4A)** and samples analyzed separately for sequencing run 1 and run 2 **(S4B)** using the 5% relative abundance cutoff. Table **S4C** shows the PERMANOVA output with more permissive OTU filtering cut-offs for pooled samples. Table **S4D** shows the PERMANOVA output for bacterial communities of rarefied samples to even depth of 3000 reads. Rarefaction was carried out using Qiime 1.9.1.

Table S4A: Variation in bacterial communities of butterflies across host species and families

Developmental stage	Grouping Variable	Degrees of freedom	Sum of squares	Mean squares	F model	R^2 value	p value
Larvae	Species	9	5.7459	0.63843	2.5961	0.42202	0.0001
Larvae	Family	4	1.881	0.47025	1.6559	0.15183	0.016
Pupae	Species	6	6.9766	1.16277	5.7079	0.53305	0.0001
Pupae	Family	4	5.3614	1.34034	5.5510	0.40964	0.0001
Adult	Species	9	7.3239	0.81376	3.1922	0.49766	0.0001
Adult	Family	3	2.3868	0.79559	2.2584	0.16218	0.0007
All Stages	Species	11	15.133	1.37576	5.1151	0.34253	0.0001
All Stages	Family	4	7.417	1.85424	5.8001	0.16788	0.0001

Table S4B: Variation in bacterial communities of butterflies across host species and families (across sequencing runs)

Developmental stage	Grouping Variable	Run 1		Run 2	
		P Value	R^2	P Value	R^2
Larvae	Species	0.0014	0.58	0.0001	0.52
Larvae	Family	0.036	0.27	0.0009	0.26
Pupae	Species	0.04	0.54	0.0001	0.64
Pupae	Family	0.04	0.54	0.0001	0.49
Adults	Species	0.0001	0.54	0.0001	0.57
Adults	Family	0.024	0.17	0.004	0.32
All stages	Species	0.001	0.36	0.0023	0.11
All stages	Family	0.0001	0.36	0.0001	0.22

Table S4C: : Variation in bacterial communities of butterflies across host species and families					
Developmental stage	Grouping Variable	> 20 Reads per OTU		≥0.005% relative abundance	
		p value	R^2 value	p value	R^2 value
Larvae	Species	0.0001	0.37751	0.0001	0.38619
Larvae	Family	0.0030	0.17164	0.0030	0.17793
Pupae	Species	0.0001	0.48646	0.0001	0.50226
Pupae	Family	0.0001	0.38188	0.0001	0.39317
Adult	Species	0.0001	0.46761	0.0001	0.47814
Adult	Family	0.0006	0.14813	0.0008	0.1511
All Stages	Species	0.0001	0.30099	0.0001	0.31675
All Stages	Family	0.0001	0.14107	0.0001	0.15052

Table S4D : Variation in bacterial communities of butterflies across host species and families post rarefaction			
Stage	Grouping variable	P value	R2 value
Larvae	Species	0.0001	0.36619
Larvae	Family	0.0030	0.16861
Pupae	Species	0.0001	0.47496
Pupae	Family	0.0001	0.37352
Adults	Species	0.0001	0.47436
Adults	Family	0.0007	0.16845
All Stages	Species	0.0001	0.29441
All Stages	Family	0.0001	0.13827

Table S5: Variation in bacterial communities of butterflies across host species and families: Table S5A shows the output of PERMANOVA (10,000 permutations) when variation in bacterial community structure was tested across butterfly species and families, using phylogenetic distance metric (weighted unifrac). R^2 value indicates % variation explained. Table S5B shows the total variation explained by the first two principle coordinates of unconstrained PCoA.

Table S5A: Variation in bacterial communities of butterflies across host species and families (PERMANOVA output)

Stage	All OTUs				OTUs with 5% abundance cut-off				Core OTUs			
	Species		Families		Species		Families		Species		Families	
	R^2	p	R^2	p	R^2	p	R^2	p	R^2	p	R^2	p
Larvae	0.354	0.001	0.195	0.005	0.357	0.005	0.186	0.014	0.393	0.003	0.258	0.001
Pupae	0.593	0.0001	0.498	0.0001	0.563	0.0001	0.451	0.0001	0.662	0.0001	0.492	0.0001
Adults	0.429	0.002	0.167	0.022	0.416	0.001	0.018	0.173	0.556	0.0001	0.189	0.035
All Stages	0.308	0.0001	0.171	0.0001	0.290	0.0001	0.0001	0.166	0.378	0.0001	0.255	0.0001

Table S5B: Total variation explained (PC1+PC2) by unconstrained PCoA analysis

Stage	All OTUs	OTUs with 5% abundance cut-off	Core OTUs
Larvae	58.52%	64.01%	66.98%
Pupae	72.61%	73.32%	82.91%
Adults	56.71%	60.51%	71.05%
All Stages	55.9%	58.93%	69.98%

Table S6: Comparing bacterial communities of larvae and their diet. The table shows the overlap between bacterial communities (comprising of the 5 most abundant bacterial OTUs) of larvae and their respective diets.

Larvae (Species)	Larval diet	OTUs shared by larvae and diet (%)	OTUs unique to larvae (%)	OTUs unique to diet (%)
A. merione	*Ricinus communis*	70	30	0
D. chrysippus	*Calotropis gigantea*	100	0	0
G. thyrsis	*Dypsis lutescens*	87.5	12.5	0
E. caudata	*Dypsis lutescens*	66.6	11	22
P. polytes	*Citrus. sp*	100	0	0
S. epeus	*Maconelicocus hirsutus*	77.7	11.1	11.1
	Average	83.6	10.7	5.5
